# Nonequilibrium *Ab Initio* Molecular Dynamics Simulation of Water Splitting at Fe_2_O_3_–Hematite/Water Interfaces in an External Electric Field

**DOI:** 10.1021/acs.jpcc.3c05119

**Published:** 2023-12-11

**Authors:** Mary T. Ajide, Niall J. English

**Affiliations:** School of Chemical & Bioprocess Engineering, University College Dublin, Belfield, Dublin 4, Ireland

## Abstract

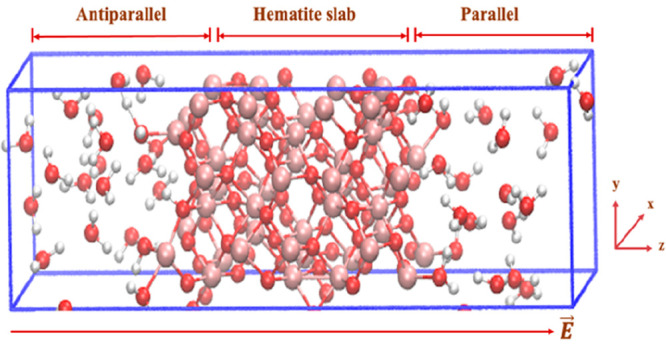

In the exploration of the optimal material for achieving the photoelectrochemical dissociation of water into hydrogen, hematite (α-Fe_2_O_3_) emerges as a highly promising candidate for proof-of-concept demonstrations. Recent studies suggest that the concurrent application of external electric fields could enhance the photoelectrochemical (PEC) process. To delve into this, we conducted nonequilibrium *ab initio* molecular dynamics (NE-AIMD) simulations in this study, focusing on hematite–water interfaces at room temperature under progressively stronger electric fields. Our findings reveal intriguing evidence of water molecule adsorption and dissociation, as evidenced by an analysis of the structural properties of the hydrated layered surface of the hematite–water interface. Additionally, we scrutinized intermolecular structures using radial distribution functions (RDFs) to explore the interaction between the hematite slab and water. Notably, the presence of a Grotthuss hopping mechanism became apparent as the electric field strength increased. A comprehensive discussion based on intramolecular geometry highlighted aspects such as hydrogen-bond lengths, H-bond angles, average H-bond numbers, and the observed correlation existing among the hydrogen-bond strength, bond-dissociation energy, and H-bond lifetime. Furthermore, we assessed the impact of electric fields on the librational, bending, and stretching modes of hydrogen atoms in water by calculating the vibrational density of states (VDOS). This analysis revealed distinct field effects for the three characteristic band modes, both in the bulk region and at the hematite–water interface. We also evaluated the charge density of active elements at the aqueous hematite surface, delving into field-induced electronic charge-density variations through the Hirshfeld charge density analysis of atomic elements. Throughout this work, we drew clear distinctions between parallel and antiparallel field alignments at the hematite–water interface, aiming to elucidate crucial differences in local behavior for each surface direction of the hematite–water interface.

## 1 Introduction

In 1976, Hardee and Bard^1^ first introduced Fe_2_O_3_ as a material for photolysis in the quest for seeking a photoanode material that was both stable under anodic polarization and capable of absorbing light of wavelength longer than 400 nm. In recent years, α-Fe_2_O_3_ has gained significant attention and is now considered to be as promising as, if not more promising than, its counterpart TiO_2_ for photoelectrochemical (PEC) water splitting due to its potential to convert 16.8% of the sun’s energy into hydrogen^2^ and also its relative ubiquity, ample availability, low cost, and reasonable ideal band gap.^3−10^ However, despite recent progress made in furthering our understanding of the titania–water interfaces^11,12^*vis-à-vis* theoretical and molecular simulation methods,^13^ the successful modeling of hematite–water interfaces is still rather challenging and indeed intricate in its procedural complexity. Notwithstanding, the study of such metal oxide–water interfaces provides the avenue where the dynamical properties of confined water molecules can be vastly explored, particularly where hydrogen-bonded molecules play key roles in stabilizing solutes via solvent interactions and in forming “cages”. To a large extent, molecular dynamics (MD) techniques have been very useful in investigating, characterizing, and classifying the structural, dynamical, and vibrational behavior of bulk and adsorbed water molecules at a variety of metal oxide surfaces; for instance, titania–water renders itself an attractive photoanode material for solar water splitting. Moreover, molecular dynamics have been explored in an effort to further understand and improve the photoelectrochemical water-dissociation process.^14−18^ In particular, the titania–water interface has enabled the exploration of hydrogen-bond kinetics, spatial distribution functions, ion adsorption,^19^ electric double-layer surfaces,^20−22^ the emergence of vibrational modes, and water–dipole orientations. Calculations based on density functional theory (DFT) of titania–water interfaces, specifically *ab initio* MD (AIMD), have reportedly been used to simulate a variety of surfaces ranging from partial to full coverage and multiple water layers.^9,23^ AIMD has also contributed to providing key insights into librational motions of higher-frequency modes of the titania–adsorbed water interface. In fact, the effect of defects of the anatase (101) surface on the adsorption energies of water has been probed at defective sites,^24^ in addition to hydroxide ions that have been studied at these surfaces.^25^ Furthermore, Car–Parrinello MD (CPMD) simulations of pristine and defective anatase (101) surfaces have *ipso facto* been carried out with the adsorbed water layers. Other CPMD work performed has also led to different surface reconstructions of rutile (011) aside from those under vacuum or low-coverage conditions. In terms of the hematite–water interface, the study of the rate of water breakup events *vis-à-vis* nonequilibrium *ab initio* molecular dynamics (NE-AIMD) simulation under the effect of an external applied static electric field and electromagnetic (e/m) fields has been predicted to increase by ∼70% in the linear-response regime hitherto, and the simulations performed have initiated further scrutiny of the properties of adsorbed water molecules and underlying atoms therewith,^14^ which evidently have been proven in this study to enhance the production of H_2_ in an energy-efficient manner. In addition, the transport of ionic species within the bulk-water phase also explored by the MD technique has served to enhance the processes or properties necessary for optimizing the performance and efficiency of PEC systems.^15^

**Figure 1:**
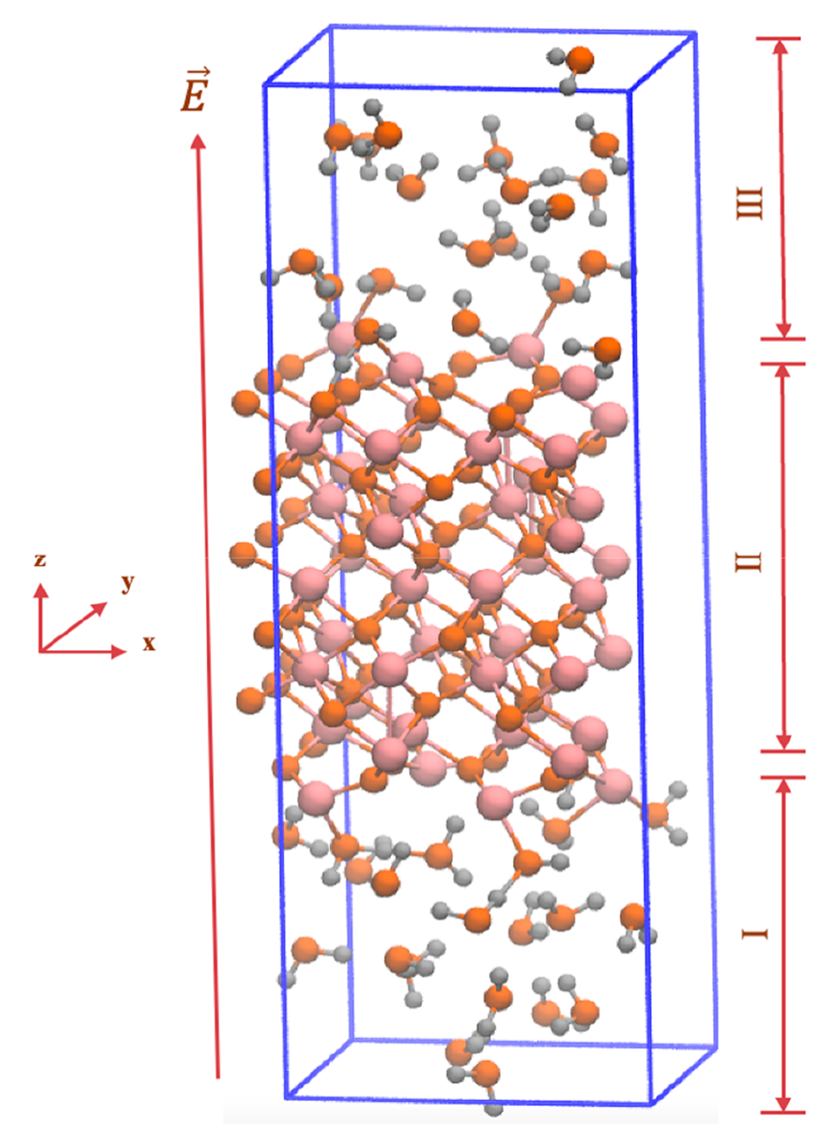
Representative models of the aqueous hematite interface implemented for the calculation. The snapshot of the supercell was performed under periodic boundary condition (PBC) after zero-field conditions equilibration. As clearly shown, the direction of the applied static electric field *E* is indicated by the dark-orange arrows pointing along the + *z* axis of the supercell. (I) The left-hand side (LHS) denotes the lower surface wherein the external-field vector is in antiparallel alignment *vis-à-vis* its local surface normal. (II) The hematite slab located in the central part of the supercell where iron (Fe) atoms are shown in pink and oxygen atoms are shown in orange and in Figure S1 in the Supporting Information they are shown in red. (III) The right-hand side (RHS) denotes the upper surface with parallel field-vector alignment. The water molecules, where oxygen atoms are depicted in orange, and in Figure S1, as red while hydrogen atoms are depicted in silver and in Figure S1 as white, fill the supercell on both sides, under which the period boundary conditions are imposed.

An intriguing and yet sparsely explored area is the application of an externally applied electric field at the hematite–water interface to boost the performance and efficiency of photolysis. Aside from electrolysis, the production of H_2_ from water *vis-à-vis* electric fields has received significant attention in instances such as ionization^26^ and plasma methods.^27^ The application of explicit external applied electric fields to boost the light-driven photoelectrochemical water splitting is still rather limited in the literature, although notable work has been performed.^28^ Clearly, the use of *ab initio* MD (AIMD) in conceptualizing the *modus operandi* ascribed to the application of external electric fields enhancing water splitting is paramount to this study and as such is depicted in Figure 1, which posits that under light irradiation and within an appropriate frequency range (where the band gap is exceeded by the photon)^29^ an electron is promoted to the conduction band, thus creating an electron–hole pair. In most metal oxide–water interfaces, there exists hydroxylation due to the (partial) chemical adsorption of the water (also “in the dark”) for considering hematite^8^ and titania,^30−32^ while a water molecules becomes “strained” as it breaks up due to the hydroxylation of a bridging atom at the hematite iron oxide surface. Nonetheless, upon application of an electric field, the hole and electron drift in opposite directions as a result of their opposing charges as seen clearly in Figure 1 (wherein the applied static electric field are respectively seen to be pointing along the +*z* axis for both supercells), where evidently the electric field breaks symmetrically^33^ in order to pave the way for the enhancement of electron–hole transport.^8,33,34^ In fact, the enhanced drift combined with increased diffusion due to the oscillating nuclei in the external field substantially attenuates the obvious problem of electron–hole recombination and, essentially, helps to enhance the contact of the holes with water at the surface, thus facilitating water breakup. Moreover, external electric field water molecule dipoles are inclined to align partially with the applied field, in which for alternating (e/m) fields the water dipoles rotate back and forth with the applied field as it changes direction,^35^ thus leading to substantial intramolecular strain and actually further enhancing the rate of the water splitting. Perhaps this can serve to intensify further chemical adsorption of hydrogen onto bridging oxygen atoms at the surface, although this is dependent on a balance between enhanced drift in the lower-frequency field and optimal overlap of external-field frequencies with reorientation of the dipole alignment usually in the microwave region.^35,36^ However, given the aforementioned, there still exist limited AIMD studies on the hematite–water interface in comparison to the titania–water interface, despite recent progress^37,38^ and the promising prospect of iron oxide for catalytic reactions and water splitting.^39^ Encouragingly, Bergermayer et al.^40^ and Rohrbach et al.^41^ have successfully reported the used of DFT to characterize the structure and composition of these dry surfaces, in addition to the experimental evidence of 001-surface stability.^40,41^ Trainor et al.^42^ and Yin et al.^43^ also used a DFT study for the adsorption of water on hematite surfaces, which are in close accord with the experimental evidence of hydroxylated termination being more stable.

Of utmost importance to the present study is the application of nonequilibrium AIMD (NE-AIMD) to investigate the influence of externally applied electric fields of varying magnitudes on the α-Fe_2_O_3_/water interface. Bearing in mind previous seminal work performed on similar hematite/water interfaces in electric fields,^44^ this prompts many unanswered questions, such as determining the coordination number of the atoms and molecules or ions involved in the splitting process of the interfacial system; the observed correlation among the hydrogen-bond strength, bond-dissociation energy, and H-bonds’ lifetime, and the Hirshfeld charges described in relation to the deformation density and the differences between the molecular and unrelaxed atomic charge densities, in addition to gleaning rigorously at the structural and dynamical motif at the surface, as well as the emergence of vibrational and librational modes and atomistic surface contours and shapes corresponding to the hematite–water interface. Indeed, in the current study, we are interested in a longer time scale of the AIMD simulation while investigating the effect of increased intensity on structural and dynamical properties describing in detail the intricacies of the hematite–water interface and the water-splitting process. To accomplish that, AIMD is further applied to this study considering its rigor in capturing the rich physical complexity of room-temperature chemical adsorption of water molecules to the surface hematite. In addition to the relatively low barriers required for water dissociation reported by Nguyen et al.,^45^ this water-dissociation event can possibly be observed over picosecond time scales in room-temperature AIMD. In any event, the current study seeks to further probe the propensity of water splitting under the effect of a static field of increased magnitude 0.10 V/Å, in comparison with 0.05, 0.075, and 0.0875 V/Å and while addressing, *inter alia*, the simulation of the α-Fe_2_O_3_ (001) surface in contact with physical adsorbed bulk layers of water.

## 2 Computational Methodology

Nonequilibrium *ab initio* MD (NE-AIMD) simulation was performed on water in contact with hematite-(001) Fe_2_O_3_ surfaces under three-dimensional periodic boundary conditions (PBC). For initial system relaxation prior to DFT, the SPC/E water model^46,47^ in the form of the condensed state of liquid water was placed in contact with two periodically imaged surfaces of a mobile slab of pristine, initially anhydrous α-hematite. From the bulk portion of the α-hematite slab, the surface was cut into a 2 × 1 × 1 orthorhombic simulation cell (*x* = 10.076 Å, *y* = 8.726 Å, *z* = 13.772 Å) containing 120 atoms in order to obtain a nonpolar and dipole-free surface. The direction of heterogeneity was on the *z* axis. At a density of ca. 1 g/cm^3^, 42 liquid-state water molecules were kept in contact with the hematite with a similar *x* – *y* simulation box and a water-layer system thickness of ca. 13 Å. The SPC/E water layer used allowed for the layer in contact with hematite to be adsorbed physically via a motif from molecular mechanics energy optimization set at 0 K, in addition to a rigid hematite slab with charges of +1.5*e* on the iron ions and −1*e* on each oxygen ion. The DFT used originated from the vdW-DF function with an explicit nonlocal Dion–Rydberg–Schroder–Langreth–Lundqvist (DRSLL) correlation correction to describe the van der Waals interaction.^48,49^ An optimized Becke 88 (optB88) generalized gradient approximation (GGA)-type functional^50^ was initiated for the exchange part. The DRSLL approach was selected due to its well-defined description of water molecules and dynamic characteristics,^51−55^ particularly in a nonequilibrium simulation influenced by electric fields.^56^ Given the acceptably large size of the periodic system and water layer in the *z* direction, Γ-point sampling was applied. After convergence checks, the plane-wave cutoff energy was set at 400 *Ry*. Before AIMD and after molecular-mechanics optimization, partial optimization and lattice parameters were used with the VdW-DF method until atomic forces attained a value smaller than 0.02 eV/Å. Nguyen et al.^45^ made an improvement in the quantitative DFT simulation of hematite–water interfaces via GGA+U that allows for water dissociation of lower-energy barriers. Since the main focus of this study is predicated on exploring the basic mechanism of the influence of fields on water dissociation and particularly if the underlying (zero field) dissociation energy barrier is lower (such as with PBE+U^45^), difficulties will arise when differentiating field-mediated intramolecular dissociation from those intrinsically present within the GGA+U framework’s lower energy barriers. Hence, with a certain level of hesitation, vdW-DF was chosen with the exception of the Hubbard U term for this study in order to allow for a concise mechanistic distinction between field-induced breakage and underlying dissociation.

In order to set apart athermal effects to a feasible extent from thermal effects, nonequilibrium (N)NVT was performed for the propagation of NE-AIMD in the external electric fields. Under the field effects, second-generation Car–Parrinello MD^57^ was initiated for at least 50 ps NE-AIMD trajectories, since second-generation Car–Parrinello MD^57^ reportedly gives a good indication of the structural and dynamical properties of water in terms of the radial distribution functions, vibrational spectra, and self-diffusion function. A time step of 1 fs was initiated alongside the Langevin stochastic thermostat for NE-AIMD, followed accordingly by zero-field equilibrium AIMD.^57,58^ In-house-modified CP2K software^56,59^ was initiated for NE-AIMD, and the DZVP basis set with core-electron GTH pseudopotentials was implemented for the wave function from the CP2K database. The Berry-phase approach was implemented for the imposition of uniform static electric fields *E* applied along the laboratory +*z*-direction axis as shown in Figure 1(60) with field strength/intensity values of 0.05, 0.075, 0.0875, and 0.10 V/Å. Considering that in the condensed phase water exhibits intrinsic electric-field intensities in the range of ∼2 to 3 V/Å,^35,61,62^ the external-field torque on each molecule is on the order of 2–5% of those inherently present due to interaction with neighboring molecules, which evidently affords an appropriate signal-to-noise ratio and transition into the nonlinear response of regime at ∼0.05–0.1 V/Å.^62^

As previously explained, DFT was implemented in the simulation of aqueous hematite surfaces with periodic boundary conditions imposed in all three dimensions. The supercell structure of size 9.743 × 8.438 × 27.023 Å^3^ containing 24 Fe_2_O_3_ units in the hematite-(001) slab and 42 water molecules was applied. Furthermore, in the absence of an applied external electric field (i.e., zero-field condition), the supercell structure accommodates an equilibrated model structure from the 100 ps equilibrium AIMD structure. At the onset of the simulation process, equilibration was performed on the system from its initial structure that was first optimized and afterward heated to a temperature of 330 K that is normally used to simulate room-temperature properties of liquid water within DFT.^56,63^ Following up from this simulation stage involved the use of Langevin thermostat parameters that were optimized with the ASPC corrector^64^ in the second-generation CPMD procedure.^57,58^ In conjunction with the long 1 fs initiated and in the absence of any reactive force field,^65^ Langevin parameters were applied alongside to ensure numerically stable simulations. This configuration was subsequently employed in the study of properties such as structural, dynamical, vibrational, and librational motion, the atomistic surface contours, and shape under the applied external electric field effect in conjunction with AIMD simulations under both equilibrium (describes as zero field) for at least 100 ps and in-field (NE-AIMD) conditions for a minimum of 50 ps.

## 3 Results and Discussion

### 3.1 Water Adsorption and Dissociation

**Figure 2:**
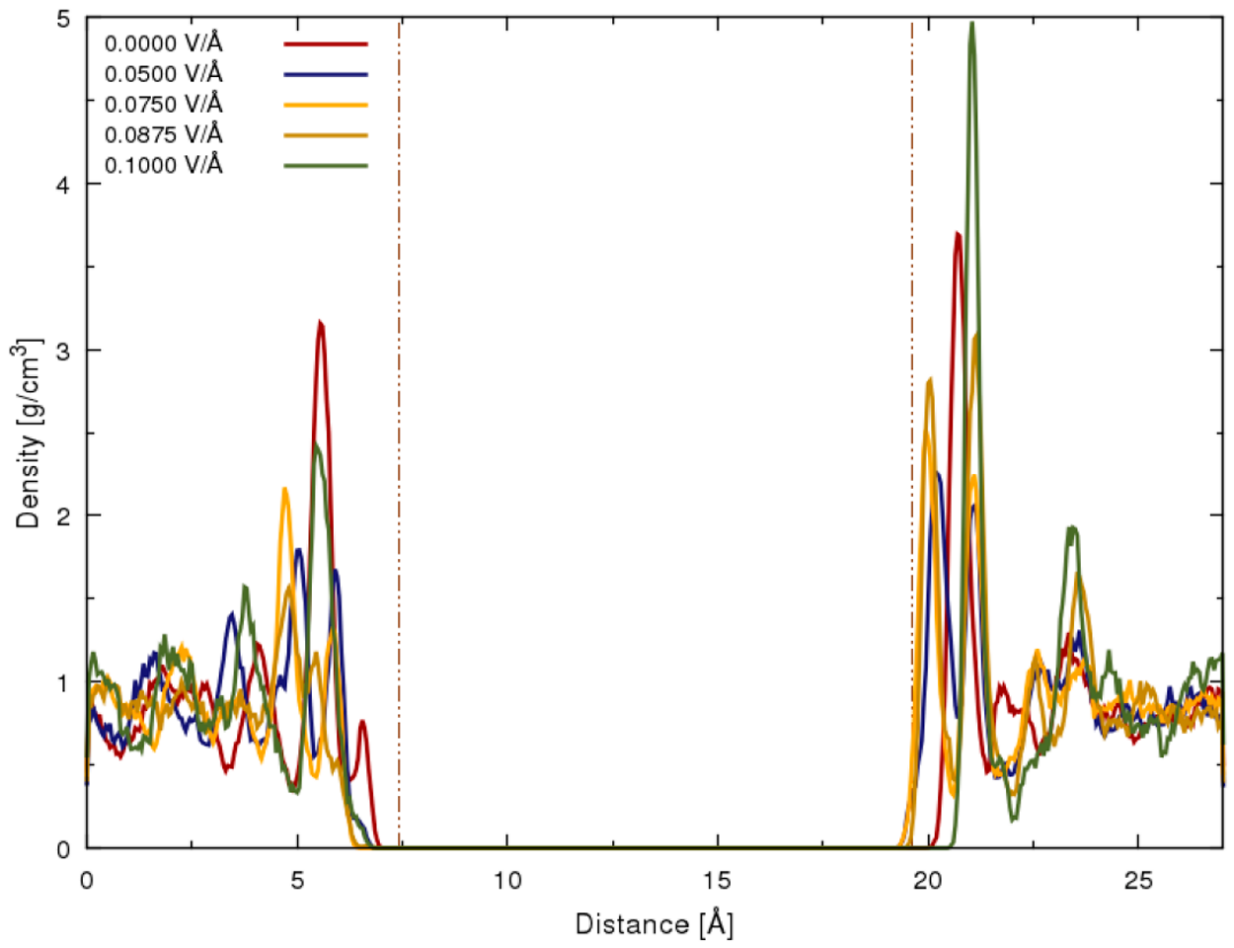
Density profile of water molecules of the oxygen (O_W_) atoms in the interfacial or adsorbed water region of the hematite. The zero-field condition and profile are affected by the static field of magnitude 0.10 V/Å applied in the direction perpendicular to the hematite surface. The vertical dotted–dashed lines represent interfaces with surface direction alignment as depicted in Figure 1 denoting the left-hand side (LHS) as the lower surface wherein the external-field vector is in an antiparallel alignment *vis-à-vis* its local surface normal and the right-hand side (RHS) denoting the upper surface with parallel field-vector alignment. Static field intensities of 0.05, 0.075, and 0.0875 V/Å are added for appropriate comparison.

**Figure 3:**
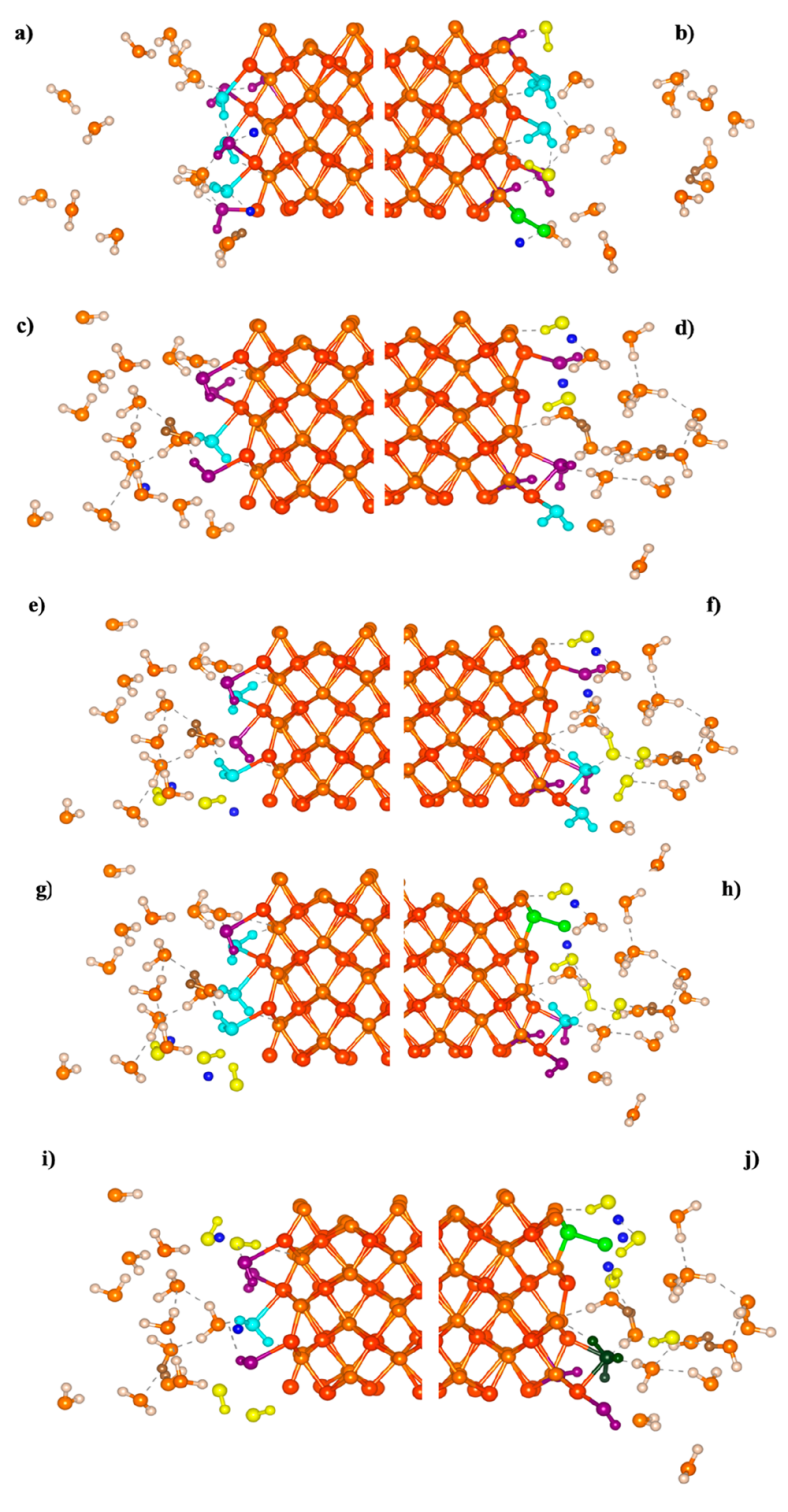
*Ab initio* molecular dynamics (AIMD) snapshots of the aqueous hematite interfaces showing evidence of the adsorption and dissociation–diffusion mechanism in addition to the early onset of covalent-bonded types occurring between H atoms. The applied static electric field *E* is oriented along the +*z* axis of the supercell (cf. Figure 1). As shown, hematite slabs are located at the center of the supercell where Fe atoms are shown in red and oxygen atoms (either in the hematite or water) are shown in orange while the surrounding water molecules are shown in yellowish-white. (a) Zero-field condition after 22.3 ps of the AIMD production equilibrium with the antiparallel external-field alignments (i.e., LHS) represented in (c) a static electric field of 0.05 V/Å magnitude at 26.4 ps; field of 0.075 V/Å magnitude at 25.2 ps; (g) field of 0.0875 V/Å magnitude at 30 ps; (i) field of 0.10 V/Å magnitude at 30.3 ps and the parallel external-field alignments (i.e., RHS) represented in b, d, f, h, j. In all cases, H_3_O^+^–H_2_O–OH^–^ entities are clearly visible with the adsorbed hydronium ion, hydroxyl groups, and nondissociated water molecules highlighted respectively in dark-green, purple, and cyan while oxygen adsorbed to the surface, dissociated hydroxyl groups, and hydrogen protons are respectively depicted in light-green, yellow, and blue. In addition, covalently bonded contacts between H atoms are shown in brown.

To demonstrate clearly the structural properties of the layered surface of the aqueous part of the interface, it is appropriate to first present the density profile (cf. Figure 2) of the water molecules obtained from the distribution of the water oxygen (O_w_) atoms in the direction perpendicular to the hematite surface (*z* direction in laboratory system axes). In light of our previous study,^44^ the density profile was obtained with a shorter time scale and the omission of the increased field strength of 0.10 V/Å. Here, we clarify in detail the width of the interfacial region with respect to zero-field and static-field conditions while fulfilling previous studies’ unanswered questions regarding the adsorption and dissociation of water in the interfacial region. The distribution predicated on the distance of the water-oxygen (O_w_) profile in the interfacial region exhibits a well-defined peak associated with the adsorbed-water layer on the surface. The reference point of the density distribution similar to that in previous studies^66^ was selected as the mean position of the topmost Fe atom layer on the corresponding surface. As depicted in Figure 2, the boundary of the interfacial region indicated by a vertical-dashed line was chosen as the last significant minimum for both profile surface directions before becoming flat in the bulk-water region. The presence of the adsorbed water region in either surface direction from hematite oxide atoms allowed for the proper sampling of the respective properties in each consecutive layer. The distance of the interface region is examined to range between 6.5 and 7.0 Å for concomitant antiparallel alignment and surface distance observable at ca. 25 Å for the parallel alignment. An indicative fact shown by the first observed peak in the zero-field condition of O_w_ highlights the presence of the physisorption process whereby water molecules from the first solvation layers rapidly adsorb chemically to nonsaturated surface iron atoms despite the initial configuration constructed in such a way that the first adsorbed layers of the hematite surface were made to interact weakly with metal oxide. Moreover, the appearance of oxygen and three surface Fe atoms at the start of the DFT simulation initiated stronger ionic bonds, thus enabling the intensified electrostatic force. In the equilibrium state, while in the absence of no field, both parallel and antiparallel alignments observe on average the onset of four water molecules chemisorbed on both hematite surfaces alongside the spontaneous rapid dissociation of H^+^ and OH^–^ ions (cf. Figure 3). Due favorably to the nucleophilic nature, hydrogen protons (H^+^) migrated to the closest surface oxygen atoms while the negatively charged hydroxyl ions (OH) are seen to remain firmly attached to the surface iron, as shown vividly in Figure 3. On the hematite surface, two distinct behavioral pattern are known to be exhibited by hydroxides and protons, while the mobility of protons is accompanied by an intricate binate form and subsequently changes in pattern from the outward or inward direction due to their small size, which evidently affects the degree of surface acidity,^67,68^ whereas hydroxyl groups are more restrictive in mobility by the surface interaction and considerably by strong hydrogen bonds in the first two adsorbed layers. Essentially, during the onset of equilibrated simulation and preceding the stable structural form, extensive time precisely (beyond ∼0.6 ps) increases the propensity of the hydronium (H_3_O^+^), particularly under the applied electric field effect (cf. Figure 3), to become attracted to the hematite surface oxygen atom, hence causing it to donate one of its protons to form chemical bonds. However, the occurrence of hydronium (H_3_O^+^) in the water-splitting process was unresolved in a previous seminar study by the author of ref (44). In this study, we further elucidate the inception of initial, partial hydroxylation of the hematite surface, *ipso facto*. Proceeding with this, the structure remained stable (after ∼0.6 ps) due to it attaining formal hydroxylation with proton and surface alteration by physicochemical electrostatic bonds with either the water molecules or diatomic anion (OH^–^) that are involved in the exchange of protons with surrounding water molecules (cf. Figure 3). Moreover, possibly after an elapsed time (ca. 2 to 3 ps), increased dipolar alignment of the water molecules enhanced the proclivity to break and reform with oxygen atoms at either extreme side of the hematite surface as shown clearly in Figure 1, in which at a more extended time, appropriate reorientation of water molecules occurred from the initial bulk-like configuration.

Static electric fields of magnitudes 0.05, 0.075, 0.0875, and 0.10 V/Å were applied to the equilibrated system in the direction perpendicular to the hematite surfaces as shown in Figure 1. As expected, in the absence of an electric field (zero field) there exists no dipole-orientational preference, and in promulgating the system *vis-à-vis* NE-AIMD, it is also anticipated that the partial dipole-alignment response of the water molecules occurred in conjunction with the required concomitant reorientations also observed by the study performed^56^ reporting intensified dipole alignment and locking with greater field strength or intensity.^36^ At the hematite surface, the presence of electric fields is met with the occurrence of a disturbance (e.g., perturbation) that affects the force balance existing between the surface and its corresponding first solvation layer, thus disrupting the interfacial water network that is held together by a considerably strong hydrogen-bond network and electrostatic interactions that inevitably accelerate the water-splitting reactions. Moreover, these observations are strongest when the external applied fields are efficiently applied toward the direction of the hematite surface as shown in Figure 1, where the external-field vector points toward the Fe_2_O_3_-oxide slab in the antiparallel field alignment, which differs from the applied field in the direction parallel to the outward surface normal. Notably, the difference observed for both surface field directions is common in all NE-AIMD and remarked to perhaps contribute greatly to the study of rich and vital differences in the local behavior of both distinctive interfaces. Nonetheless, the presence of an external electric field serves importantly as a contributing factor in defeating the presently low energy barriers associated with the water-splitting process in that the protons accompanied by the water molecules are galvanized toward greater field-induced and highly reactive surface oxygen atoms. In addition to experiencing a series of proton jumps from the newly formed hydronium ion to the next hydrogen-bonded neutral molecules along the field direction, this continues to form another hydronium ion from which different protons continuously “hop and jump”. In spite of electric-field corroboration boosting the water-splitting process and indeed solar to hydrogen production, evidence of the inception of surface- and field-induced H_2_ formation particularly informed by the constant migration of protons via “Grotthuss hopping” in both surface directions is shown clearly in Figure 3 and is evidently more pronounced in the parallel alignment direction (i.e., upper surface) of hematite slabs. Moreover, perceptible contact is likened to the covalent-bond type and exists between hydrogen atoms that form moieties analogous to H_2_ molecules, in which such formations are either close in similarity to independent H_2_ molecules or part of entities where two protons share the same crystal structure of the oxygen atom as noted in a previous study.^14^ Nonetheless, incipient covalent-bond-type H–H contact initiated by water–proton and water–water contact (cf. Figure 3) allows for suitable water dissociation and an increased propensity for nascent H_2_ formation that is facilitated by the local intrinsic electric-field intensity in close proximity to the hematite surfaces.

Quite evidently, the density profile (cf. Figure 2) discloses the release (electro-) of protons from water that are remarked to be stabilized on the negatively charged hematite surface, especially for antiparallel external-field alignment. However, the interaction of the hydroxyl group with iron (Fe) is weakened due to the applied electric field that causes the protons (in contrast to the entire parent water molecules to which they primarily belong) to be pulled toward the bulk-water region. Subsequently, these proton-drift effects are displayed considerably in changes in water-oxygen (O_W_) atoms (density profile) that are in close proximity to the solid surface (where the vertical dotted–dashed lines separate the interfacial region of the hematite affected by the electric field), as evinced from the distance shifting which causes the reorientation of the water molecules in the antiparallel-field application. Nevertheless, evidence of ensuing hydroxyl chemisorption to the nonsaturated surface Fe atoms is strong and thus exhibits a proclivity to hinder desorption. Of apparent noticeably stark difference is the electric-field response in terms of the adsorbed water layer surface. Considering the upper surface (i.e., parallel field-vector alignment *vis-à-vis* its local surface normal), the applied field also acts to stabilize the dipole moment magnitude of the first adsorbed layer, while for the lower surface (i.e., antiparallel alignment), the applied-field torque reorients the water molecules at the interface, which in turn considerably weakens the hydrogen-bonding interactions with the surface and causes partial desorption and lesser surface depolarization, in close agreement with reactive and empirical studies on TiO_2_ water interfaces.^38,61^ These observations are also likened to the changes in the lengths and angles of hydrogen bonds between the surface oxygen atom and water of the hematite–water interface shown in Figures S6 and S7 in the Supporting Information.

### 3.2 Surface–Water Interactions

**Figure 4:**
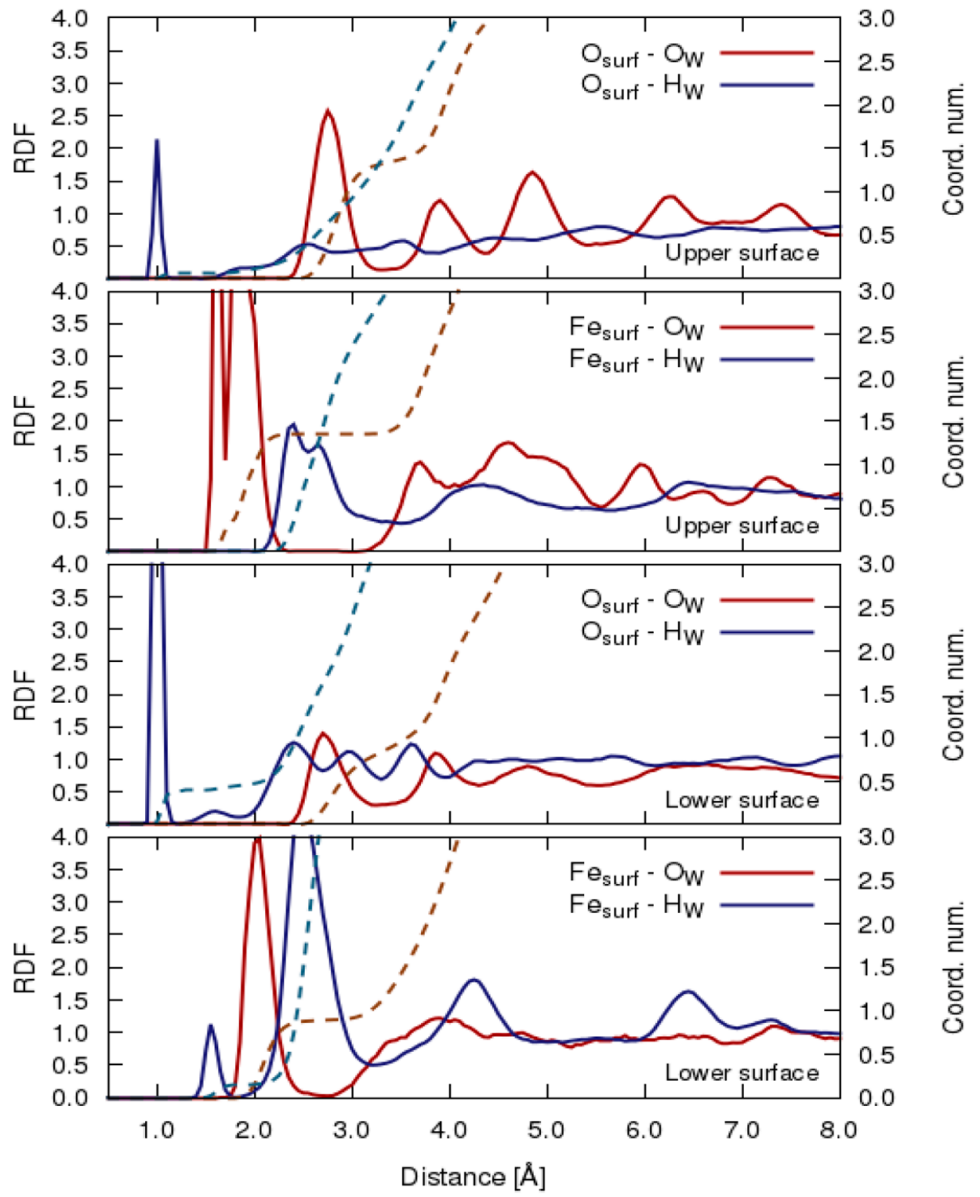
Radial distribution functions (RDF) detecting the interaction of hematite surface iron (Fe_surf_) and oxygen (O_surf_) atoms with hydrogen (H_W)_ and water oxygen (O_W_) under an external static electric field of 0.10 V/Å for both surface direction alignments as depicted in Figure 1 denoting the left-hand side (LHS) as the lower surface wherein the external-field vector is in an antiparallel alignment *vis-à-vis* its local surface normal and the right-hand side (RHS) denoting the upper surface with parallel field-vector alignment.

To probe further the effect of an applied field on the surface/water interaction of the hematite–water interface, the radial distribution function (RDF) and coordination number were calculated for the static electric field with magnitudes of 0.05, 0.075, 0.0875, and 0.10 V/Å to obtain an accurate comparison with the zero-field condition (cf. Figures 4 and S2–S5 in the Supporting Information). The coordination number was studied in detail in this current study to provide vital information on the surface water interaction taking place at the hematite–water interface, which was unexplored in a previous study.^44^ Unsurprisingly, in terms of the zero-field condition (cf. Figure S2) detecting the interaction of oxygen and hematite with water molecules for both concomitant surface alignments, the interaction of surface iron (Fe_surf_) with the first hydration layer is much stronger than that of the oxygen surface atom, indicating that surface-oxygen (O_surf_) atoms do not interact directly with the first adsorbed layer. The contact distance of these atoms with water is set at 2.5 Å in excess, which signifies that the protons released from the water are stabilized on the hematite surface iron. These are also indicative of the fact that regardless of the initial structure prepared in such a manner that the first hydration layers of the hematite surface were made to weakly interact with the metal oxide. Indeed, the first observed peak evinced a sorption process wherein water molecules from the first hydration layers adsorb chemically to the nonsaturated surface iron atoms. These are observed in the density profile (*vide supra*, Figure. 2) of the water molecules of the oxygen atoms for the zero-field condition. Notably, the closer contact of the water molecules (or hydroxyl group, OH^–^) with iron (Fe) for both surface alignments indicates clearer electrostatic interactions and pronounced evidence of a hematite-induced layering effect in the water structure relative to the bulk liquid water. These layering effects are also reported for a classical MD study of the water–titania interface,^69^ where layers were observed to be formed in close proximity to the surface, and the titania slab breaks the symmetry of the local environment and introduces an electrostatic force in the immediate vicinity of the surface structure, thus resulting in the development of layers in proximity to the surface. In any event, the distance of the interfacial region shown in the density profile (cf. Figure 2) lends credence to the idea of the initiation of layering due chiefly to the stable and strong electrostatic interactions with the hematite in the immediate vicinity of the adsorbed layer and therefore effectively immobilizes these water molecules translationally and, as a result, leads to the onset of an electric double layer that penetrates the water layers amidst the two interfaces.

Nonetheless, the first peak, first minimum, and second peak of the intermolecular structure in relation to the zero-field condition emerge at distances 1.0, 1.20, and 1.72 Å, respectively, for hydrogen water (H_w_) with the surface iron (Fe_surf_). The release of H^+^ from dissociated water is shown to reside on surface oxygen as evinced by the O_surf_ – O_w_ peak position at 1.0 Å. The third peak located at 2.5 Å relates to the hydrogen-bonding interaction of the molecular water/hydroxyl groups from the first adsorbed layer with the surface oxygen. Moreover, the integral of the surface oxygen and water oxygen interaction second peak position in the upper surface of the hydration layer emerged with a coordination number of 1.0 corresponding to the complete coverage of O_surf_ by a proton (H^+^). Evidently, water molecules chemisorbed on the hematite surface, thus leading spontaneously to the dissociation of the water molecules to H^+^ and OH^–^ species, which then resulted in the hydrogen proton migrating to the nearest surface oxygen corresponding to a higher coordination number while the negatively charged hydroxyl group remained attached to the surface iron, as also observed from the zero-field condition of the density profile. A similar observation was also noted for the lower-layer surface (or antiparallel alignment) although with a slight decline in the intensity of the first peak, first minimum, and second peak of the intermolecular structure located in the lower layer surface (or antiparallel alignment). Nonetheless, similar to the upper surface, the integral of the first peak gave rise to a coordination number of unity. Furthermore, the hydroxyl group (OH^–^) attached to the surface iron (Fe_surf_) presented itself as the first peak for upper and lower surfaces at a distance of 2.0 and 2.04 Å in the site–site radial distribution functions (RDFs) of Fe_surf_ – O_w_ following accordingly with the first peak distance of Fe_surf_ – H_w_ located at 2.5 Å for both surface alignments. In addition, the coordination number of the surface oxygen atom is also seen to be manifested at 0.50 and 0.61, respectively, for water oxygen and hydrogen atoms in both concomitant surface alignments, which could be presupposed to the structure stable *a priori* with hydroxylation and proton and *de facto* modification of the surface physicochemical ionic or electrostatic bonds with either water molecules or in the case of the hydroxyl group (OH^–^) for a stronger Fe–O ionic bond. Perhaps it could also be remarked that the coordination numbers of the O_surf_ on both ends of the surface slab are indeed covered with hydroxyl groups while the (OH^–^) groups produced by the dissociative adsorption of water are located in the first adsorbed layer and subsequently interact with the surface oxygen atoms and closest water molecules by hydrogen bonding.

Upon application of the static-field effect, the perturbation of the intermolecular structure becomes dramatically more stark with increasing field strength. Similar to the zero-field condition, the first peak, first minimum, and second peak are located at distances of 1.0, 1.20, and 1.72 Å, respectively, for an intensity of 0.05 V/Å (cf. Figure S3 in the Supporting Information). In contrast to the zero field, the first peak corresponding to the interaction between the surface oxygen atom and hydrogen water (O_surf_ – H_w_) declines in intensity with a narrowed tip at the upper surface (parallel alignment) while an increased peak intensity emerges for the case of the lower surface (antiparallel alignment). This evidently implies that the application of a field (0.05 V/Å) has indeed resulted in the weakening of the hydrogen-bond interaction on the parallel surface. This observation hitherto led to water molecules been pushed closer to the hematite surface, as evinced by the slight upward movement of the second minimum located at 2.10 Å. As expected, in comparison with the hematite surface iron, for both concomitant surface alignments, the surface oxygen atoms do not interact directly with the first hydration layer as shown by the contact distance appearing at 2.5 Å, likened also to the zero-field condition. Moreover, the proton (H^+^) released from the dissociated water resides on O_surf_ and is clearly shown by the (O_surf_ – H_w_) peak position situated at 1.0 Å for both surface alignments. This observation is a confirmation of earlier speculations made of protons (electro-) released from water that subsequently become stabilized on the negatively charged hematite surface, particularly for the antiparallel induced electric field, and the interaction of the hydroxyl group with iron (Fe) that becomes weakened as protons are galvanized toward the bulk-water region by the applied field. This proton-drift effect depicted by early detection of the interaction between surface iron and water molecules is seen to exhibit dramatic changes in the radial distribution function with increasing field intensity (cf. Figures 4, and S4 and S5 in the Supporting Information). Furthermore, evidence of the hydroxyl group attached to surface iron (Fe_surf_) is seen to be initiated by the RDF peak positions of Fe_surf_ – O_w_ and Fe_surf_ – H_w_ at ∼2.1 and 2.5 Å for both surface direction alignments. Interestingly, the surface hematite iron is seen to integrate to a coordination number of 0.64 in the lower surface while the upper surface integrates to 0.5 and 0.65 for surface iron with oxygen and hydrogen water, respectively, in addition to the second peak position interaction between surface oxygen and the water oxygen coordination number of unity corresponding to full coverage of surface atoms by H^+^. In any event, 65% (i.e., corresponding to coordination number of 0.65) of the total surface hematite iron emerged *grosso modo* on both slab ends to be covered by hydroxyl (OH^–^) groups while the rest of the (OH^–^) groups produced by the dissociative adsorption of water interact with the surface oxygen atoms and closest water molecules by hydrogen bonding.

With increasing field intensity (i.e., 0.05–0.1 V/Å), it becomes apparent, especially in the parallel surface, that the presence of an increased applied field strength perturbs the force balance between the surface and first hydration layer, which serves to distort and disrupt the hydrogen-bond network, thus accelerating the water splitting reaction, which is vividly depicted by the two-split situated in the first peak interaction of the surface hematite iron with oxygen water. Subsequent weakening of the hydrogen-bond interaction by the applied field also resulted in the water molecules being pushed further away from the interface, as evinced by the first peak divided split distance from 1.85 to ∼2.03 Å. Similar electric-field effects on adsorbed water with TiO_2_ surfaces were also reported.^61^ Furthermore, electric-field effects are pronounced as a result of the effective application of the external electric field toward the hematite surface, as shown in Figure 1, which is in good agreement with *ab initio* simulation of the electrified air–water interface where static and homogeneous electric fields applied parallel to the air–water surface plane enhanced proton transfer and the water dissociation process.^70^ Unsurprisingly, considering the field intensity of 0.075 V/Å as shown in Figure S4 of the Supporting Information, there exists a strong interaction between surface iron atoms and water molecules as evinced by the contact distance of ∼2.2 Å for both surface-direction alignments. Conversely, the first intermolecular (Fe_surf_ – H_w_) peaks for both surface alignments differ slightly with the parallel surface revealing a distance of 2.65 Å, while the antiparallel-alignment case was ∼2.5 Å, which indicates that the hydrogen bonds are indeed been pushed closer to the hematite surface in the case of the antiparallel interface. With such an observed strong interaction between the surface iron and water molecules in the hydration layer, hydrogen water depicted by the second peak gave rise to a coordination number of 1.0 for both surface alignments at the interface, which clearly shows that the Fe_surf_ is fully covered by the hydroxyl group, and with greater distance, the coordination number of surface hematite iron integrates to ∼0.55 (i.e., 55% of the total Fe) beyond the boundary of the interface region on both surfaces’ alignment. Although there is a slight decline in the peak’s intensity under a field intensity of 0.075 V/Å in comparison with 0.05 V/Å, the proton (H^+^) released from the dissociative water effect is clearly residing on the surface oxygen atom and is subsequently indicated by the (O_surf_ – H_w_) peak position at 1.0 Å for both surface alignments. In addition, the occurrence of the dissociation of water molecules is depicted by the peak position at 2.5 Å, which relates to the onset of the interaction of water molecules with surface oxygen that is preceded by the second minimal located at 2.2 Å with an upward movement (slightly larger than zero) of the hydrogen water. Clearly, the flattened peak at 2.5 Å corresponding to the hydrogen-bond interaction between the surface oxygen atom and first adsorbed layer for both concomitant alignments integrates to a coordination number of 0.60 corresponding to a coverage of 60% (on average) of the total surface oxygen atoms by (H^+^) participating in hydrogen bonding. Quite clearly, increases in the field intensity of 0.0875 and 0.10 V/Å (cf. Figures 4 and S5 in Supporting Information) also resulted in the disruption of the hydrogen-bond network, thus accelerating the water-splitting reaction, *ipso facto*. As the applied external field weakens the hydrogen-bond interaction, the water molecules are pushed further from the interface to a distance of ∼2.0 Å with the disappearance of the first halved peak (F_surf_ – O_w_) at a distance of 1.55 Å for a field intensity of 0.05 V/Å. The appearance of the first peak (Fe_surf_ – H_w_) at 1.50 Å corresponds to the hydrogen-bonding interaction of molecular water/hydroxyl groups from the first hydration layer to the surface hematite iron. It should be remarked that the sudden appearance is noticeable only for increased field intensities of 0.0875 and 0.10 V/Å. This first peak integrates to a coordination number of 1.0, corresponding to a full coverage of surface iron atoms by the hydroxyl group for a field intensity of 0.0875 V/Å, whereas for a field strength of 0.10 V/Å, the coordination number of this peak integrates to 0.80, which implies that 80% (on average) of total surface atoms participate in the hydrogen bonding. As the surface hematite iron interacts with water molecules by hydrogen bonding, the coordination number of surface iron is ∼0.65, thus on average 65% of Fe_surf_ available on both slabs participated in the hydrogen bonding and were covered by hydroxyl group for both field intensities of 0.0875 and 0.10 V/Å, whereas the rest of the (OH^–^) groups produced by the dissociative adsorption of water interacted with a surface oxygen atom in the first hydration shell of the configuration. Moreover, there exists a stronger interaction for the surface iron with water molecules as shown vividly by the contact distance of ∼2.3 Å for both field intensities of 0.0875 and 0.10 V/Å, which was further distant for the case of a surface oxygen atom with a water molecule. However, the proton (H^+^) attached to the oxygen atom is seen to manifest itself by the first peak (O_surf_ – H_w_) positioned at 1.0 Å for both slab ends. Quite clearly, the increase in field intensity between 0.0875 and 0.10 V/Å resulted in the flattening of O_surf_ – H_w_ at the antiparallel field application, which indicates evidence of applied-field torque reorienting water molecules at the interface and consequently greatly weakens the interaction with the surface that resulted in partial desorption reported also for the density profile (*vide supra*). Nonetheless, with increased field strength (*grosso modo*) of 0.075 V/Å to higher intensity, the Grotthuss hopping mechanism evidently becomes intensified with the onset of protons electroreleased from the chemisorbed water molecules in the field-parallel surface (i.e., RHS or upper surface, cf. Figures 1 and 3), and subsequently is transported instantly to the other surface (i.e., lower surface or antiparallel) via the water molecules’ hydrogen-bond network.

### 3.3 Hydrogen Bond Kinetics and Lifetime

In light of previous seminal work^44^ omitting the increased field intensity, we clarify in this study the detailed relationship existing between structural changes or the intramolecular geometry of hydrogen bonds of the water molecules at aqueous hematite interfaces with respect to those in the bulk water region. Calculations corresponding to the distribution of hydrogen-bond A–H lengths, A–D–H angles, and the number of H-bonds were obtained (cf. Figures S6–S8 in the Supporting Information). Upon exhibition of the distributions, it is necessary to first remark that the hydrogen-bond strengths were selected based on the maximum accepted distance or length between the acceptor (A) and donor (D) (i.e., A–D distance) as 3.5 Å following the Luzar–Chandler^71^ condition *ipso facto*. Clearly, the hydrogen-bond lengths vary between the systems where the hematite–water interface is seen to be subjected to increased perturbation *prima facie* in comparison to those in the water–water surfaces and bulk water region. This perhaps is unsurprising because one might anticipate that close proximity to the surface might disrupt the H-bond network and interactions, thus causing hydrogen bonds to shrink in length (and become stronger) while fighting against such an interruption. Besides, this observation is consistent under both zero field and a static field effect of varying intensity with the exception of a field intensity greater than 0.075 V/Å (cf. Table 1). This observed increment can be ascribed to the fact that increasing the field beyond a certain level inversely lengthens the bonds and inevitably reduces the strength due to reduced mutual polarization between the water molecules. Nonetheless, under all fields conditions, the donor–acceptor distances are seen to range between ca. 1.73 and 2.09 Å, wherein H-bond lengths are seen to be slightly more elongated in the upper surface (parallel field alignment) in contrast to the lower surface (antiparallel field alignment). Interestingly, it should be remarked tentatively that in certain instances the observed decrement in lengths can serve as a clear indication of stronger bond proclivity to dissociate more slowly due to higher enthalpy or energy required for water breakup.^72^ This analogy is visibly described by the H-bond lifetime (*vide infra*). Nonetheless, the observed increased length in the upper surface in contrast to that in the lower surface differs under the zero-field condition for the hematite–water interface. These are expected because the applied external field weakens the hydrogen-bond interaction in the parallel-field alignment (i.e., upper surface) as previously highlighted, in which opposite effects are noted for the antiparallel surface. This, however, demonstrates succinctly that a higher bond dissociation energy in the lower surface is due chiefly to stronger (or shorter) bonds residing in the vicinity,^66^ in addition to constant orientation of the field along the laboratory +*z* direction also lending credence to the partial dipolar alignment response of water molecules that results slightly in the lengthening of distance from zero field to static field.^56^ Perhaps, it is permissible to assert that increasing the field magnitude of water–water molecular interaction beyond a certain level could result in a contrasting effect wherein the hydrogen bond at the antiparallel field alignment becomes weakened as a result of the peak distance shifting to a slightly larger value as observed for a field intensity of 0.10 V/Å.

**Table 1:** Average Values of the Hydrogen Bond A–H Length under an Externally Applied Static Electric Field

H-bond length [Å]					
	upper surface		bulk water region	lower surface	
field (intensity)	water–water	hematite–water	water–water	water–water	hematite–water
0.0000	1.891	1.786	1.933	1.873	1.726
0.0500	1.911	1.871	1.906	1.840	1.747
0.0750	1.951	1.887	1.940	1.873	1.797
0.0875	1.925	2.042	1.907	1.871	1.846
0.1000	1.900	2.087	1.895	1.911	2.029

Furthermore, hydrogen-bond A–D–H angles examined on the basis of geometry criteria and required not to exceed 35°^56^ are displayed in Figure S7 of the Supporting Information. Similar to hydrogen-bond lengths, the angles are subjected to increased perturbation in the hematite–water interface compared to those observed at the water–water surface. Generally, the measured angles (cf. Tables 2) for both concomitant surface alignment and the bulk water region are found to range between 12.6 and 19.9°, where the angles are seen to shift to incremental values *grosso modo* (1.6–3.6°) from the upper to lower surface on the hematite–water interface while being ca. 0.2–1.5° for the water–water surface interaction. These contrasting H-bonds lengths can be tentatively rationalized as the proton drift effect highlighted in the pairwise radial distribution function, where water molecules are observed to reorient markedly in the antiparallel field application due to shorter and stronger bonds even in the absence of field effects. Moreover, the propensity of water molecules to move spontaneously in the liquid causes the disruption of the perfectly corrugated tetrahedral configuration to deviate somewhat, thus revealing the proclivity to bend in relation to the distributions.^66^

**Table 2:** Average Values of the Hydrogen Bond A–D–H Angle under an Externally Applied Static Electric Field

H-bond angle [deg]					
	upper surface		bulk water region	lower surface	
field (intensity)	water–water	hematite–water	water–water	water–water	hematite–water
0.0000	13.480	12.675	13.562	13.556	14.248
0.0500	13.347	15.254	12.923	14.213	15.171
0.0750	13.870	14.997	13.839	14.404	17.665
0.0875	13.570	15.942	13.336	15.392	17.658
0.1000	13.532	16.325	13.461	15.199	19.876

Upon exhibition of the average number of H-bonds histogram, the upper surface water interaction and bulk water region of the distribution are observed to be considerably “flattened” in comparison to the lower surface water–water interaction and hematite–water interface. Nevertheless, the average number of hydrogen bonds is greater in both flattened distributions (i.e., the upper surface water interaction and bulk region) in comparison to the elongated distributions (cf. Figure S8 in the Supporting Information and Table 3). Furthermore, the initiation of an externally applied field resulted in an increase in the number of H-bonds in the case of the bulk water region, which perhaps also indicates a strengthened polarization interaction and dipole response.^56,73^ The observed correlations of the average H-bond number with H-bond angles and lengths are quite noteworthy. In terms of the zero field and static field, the average number of H-bonds is seen to decrease from the upper to lower surface in relation to the water–water interaction, which directly correlates with H-bond lengths and inversely correlates with H-bonds angles. This inevitably implies that, in general, wider angles, a reduced number of hydrogen bonds, and shorter and stronger H-bonds are located in an antiparallel field alignment, while inversely, smaller angles, a larger number of hydrogen bonds, and longer and weaker H-bonds are situated in the parallel field alignment. It should be remarked that the initiation of an externally applied field would acts to perturb the force balance between the first surface and first hydration layer, thus irrefutably distorting (within *grosso modo* 0.05–0.1 V/Å field range) and disrupting the hydrogen-bond network, thereby accelerating the water-splitting reaction^14^ as previously highlighted (*vide supra*). Indeed, the average hydrogen-bond number in the hematite–water interface is seen to increase from the upper to the lower surface for the case of zero field and a reduced field strength of 0.05 V/Å, and moving beyond the field (e.g., 0.075–0.1 V/Å), the numbers are seen to decrease while the H-bond lengths generally decreased from the upper to lower surface for zero field and static field conditions. This can tentatively be ascribed to increasing field strengths beyond a certain level, resulting in no effect on hydrogen-bond strength, and the reverse is the case for the number of H-bonds. However, from the upper to the lower surface, it is quite obvious that broadened angles are consistent for both cases of water–water interaction and hematite–water interface, which explains the free movement of water molecules that allows for the hydrogen-bond angles to be subjected to extended bending of the tetrahedral configuration even during the dissociation process.

**Table 3:** Average Values of Hydrogen Bond Number under Externally Applied Static Electric Field

H-bond number					
	upper surface		bulk water region	lower surface	
field (intensity)	water–water	hematite–water	water–water	water–water	hematite–water
0.0000	1.820	0.039	1.554	0.371	0.085
0.0500	2.194	0.047	1.767	0.393	0.085
0.0750	1.936	0.078	1.323	0.413	0.068
0.0875	1.910	0.095	1.733	0.285	0.055
0.1000	1.977	0.053	1.920	0.229	0.031

The hydrogen-bond lifetimes were obtained using the Luzar–Chandler model of hydrogen-bond kinetics^71^, which takes into account the rate of hydrogen-bond making/breaking as well as diffusivity. Since hydrogen bonds between water molecules generally have an average lifetime of 10 ps,^74^ the calculated average lifetime or, alternatively, the persistence time (between formation and breakage) was generally observed not to exceed 10 ps. As shown in Table 4, the values were found to be 3.8–4.7, 3.5–6.8, and 0.7–8.2 ps for the bulk region and the water–water and hematite–water interfaces, respectively, which are in close agreement with a classical MD simulation study on the electric-field effect^61^ of the titania–water interface, and when compared with the titania–water interface^66^ in the absence of an electric field effect, these values persisted longer, even in the absence of an externally applied field (i.e., zero field condition). Notably, increased rates of hydrogen-bond breakage (i.e., faster decay time or a reduced hydrogen-bond lifetime) are seen to exist in the case of parallel-field alignment (upper surface). This is a very useful proposition and is expected because one might anticipate that stronger (shorter) hydrogen bonds should dissociate slowly with a longer persistence time (in relation to antiparallel field alignment–lower surface) due to increased bond dissociation energy.^72,75^ Although increasing field intensity beyond 0.0875 V/Å resulted in the opposite effect in the upper surface where a delayed decay time (longer lifetime) was noted at 0.10 V/Å, this observation was also noted for H-bond lengths reported previously.^61^

**Table 4:** Mean Hydrogen-Bond Lifetime, τ, Fitted by the Luzar-Chandler Model

H-bond lifetime [ps]					
	upper surface		bulk water region	lower surface	
field (intensity)	water–water	hematite–water	water–water	water–water	hematite–water
0.0000	3.825	0.746	3.831	6.764	1.466
0.0500	3.500	1.177	4.177	4.174	4.403
0.0750	3.501	3.498	4.138	4.368	8.173
0.0875	3.556	2.401	3.855	5.208	4.558
0.1000	4.400	3.135	4.740	3.662	3.130

As further validation, the relaxation of hydrogen bonds *vis-à-vis* the autocorrelation function (cf. Figure S9) showed visibly how the water–water surface interaction (both upper and lower surfaces) took a considerable longer time to decay in comparison to the bulk water region, and the longer time taken for the lower surface to converge to the limiting value of zero in comparison to the upper surface is quite apparently, although with the exception of the field intensity of 0.10 V/Å. Similarly, a reduced rate of hydrogen breakage (slower decay time) is seen to exist in the lower surface hematite–water interface while a faster decay time is observed for the upper hematite–water interface. These observations are unsurprising because stronger bonds situated in the lower surfaces serve as a major factor contributing to the decay time, in addition to the higher bond dissociation energy required, which in fact resulted in long-term stabilization of hydrogen bonds (involving both breakage and reformation events of water molecules) to remain at their respective positions in the layers (i.e., water–water and hematite–water), thereby allowing the required occurrence of electron transfer and importantly an increased propensity of water photolysis. At this point, one might anticipate that increasing the field intensity of the relaxation time will result in reduced hydrogen-bond time (faster decay time). Nevertheless, this is still puzzling and yet disputable due to the molecular orientation becoming almost “locked” (disclosing the dipolar “locking” effect), particularly at higher field intensity where rotational motions are suppressed^35^ with increased viscosity, reduced molecular mobility, and self-diffusivity.^76^

### 3.4 Vibrational and Librational Motion

**Figure 5:**
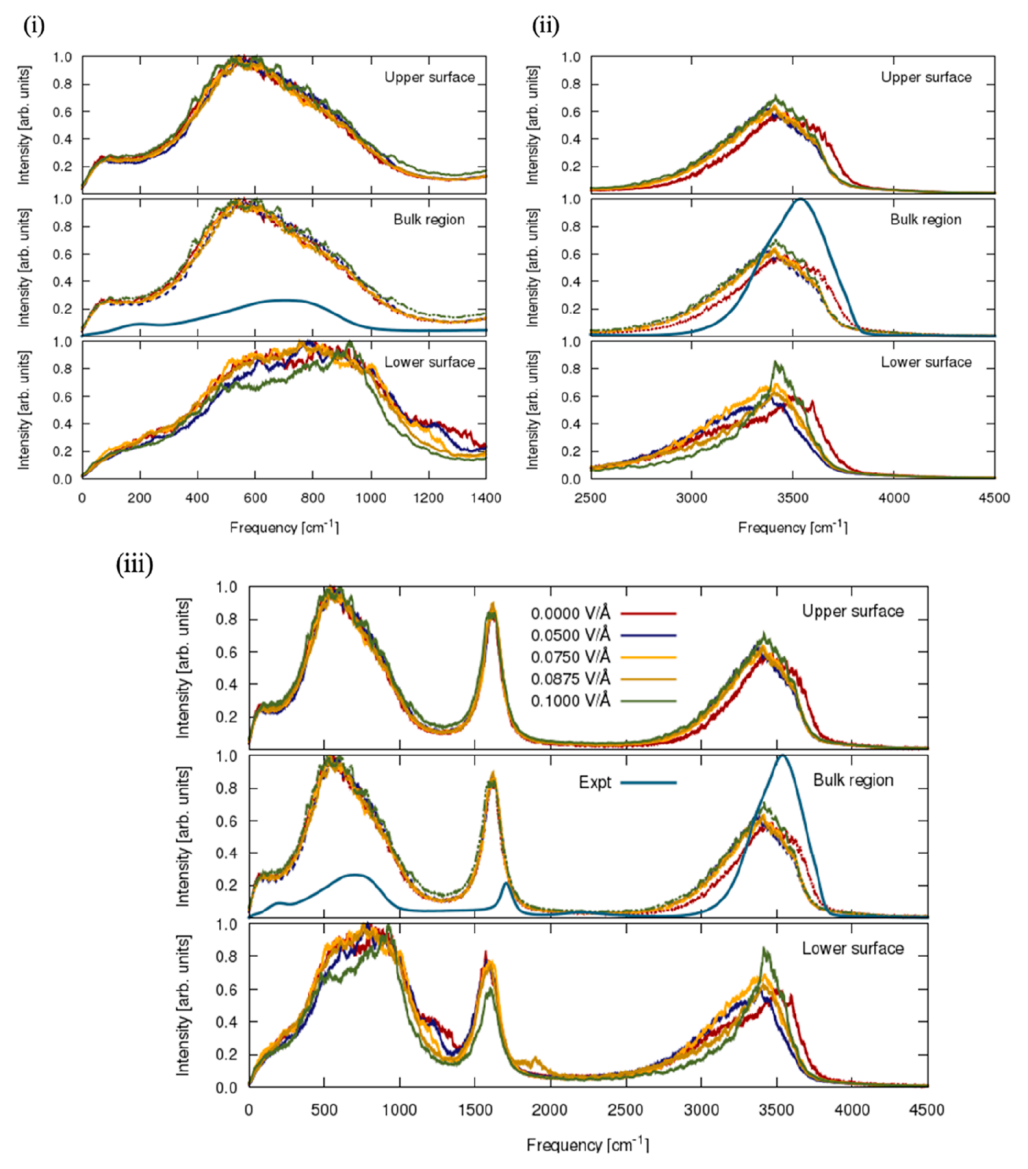
Vibrational density of states (VDOS) calculated from the Fourier transform of the velocity autocorrelation function. Effects of the static electric fields of magnitudes 0.05, 0.075, 0.0875, and 0.10 V/Å are depicted in comparison with the zero-field spectrum of magnitude 0.00 V/Å wherein (i) denotes the librational and (ii) represents the stretching bands. Attached also is (iii) the complete spectrum. The experimental curve measured in the laboratory is compared therewith. The effect of the applied static electric field in either surface direction alignment as depicted in Figure 1 denotes the left-hand side (LHS) as the lower surface, wherein the external-field vector is in an antiparallel alignment *vis-à-vis* its local surface normal and the right-hand side (RHS) represents the upper surface with parallel field-vector alignment.

The field effect on the vibrational and librational bands of IR spectra was analyzed by Fourier transforming the velocity autocorrelation function of the hydrogen atoms. The vibrational density of states (VDOS) informs an unresolved open question unexplored from a previous seminal study.^44^Figure 5 vividly shows the comparative analysis of VDOS performed between the bulk water and hematite–water interface which involved characterizing the respective collective motion of the water molecules corresponding to the librational, stretching, and bending modes. The resulting spectra of the bulk region are displayed in conjunction with experiential spectra. Quite visibly, the complete depiction of the spectra (cf. Figure 5iii) produces characteristic bands typical of a water-based system, and the laboratory curve in the plots undoubtedly mimics the experimental curve which would serve as a basis for accurate comparison. By first examining the librational modes (cf. Figure 5i), spectra in the bulk region are seen to reasonably mimic the experimental curve, although with an elevated mode, which suggests the confinement of water molecules on the hematite surface that acts in a way *per se* to disrupt or obstruct the motion of the water molecules. This trend is also likened to the upper surface in which generally striking differences in the spectral features relating to the bulk water region and adsorbed water surface are more obvious in the lower surface where the external field is applied to the hematite surface. Nonetheless, it is readily evident that the librational band with dominant peaks within the frequency range of ∼250–1200 cm^–1^ exists in the case of the bulk region and upper surface. While it is obvious in both bulk water and the upper surface region that the water molecules on the librational modes are unaffected by the applied field, detectable effects are seen to occur in the lower surface with the position of the librational modes observed to be broadened with no peak evident. This lack thereof is a result of evidence of an increased rigid hydrogen-bonded hopping pathway along which protons conduct superionically via the Grotthuss mechanism,^77^ especially at higher field intensity with a minimal avenue for rotational and oscillating motion caused by confinement and suppression effects. Nonetheless, the evidence of “wagging modes” is seen to be more pronounced in the frequency range between ca. 400 and 1400 cm^–1^ for the lower surface, which confirms the presence of the confinement of hydrogen atoms/protons normal to the hematite surface also observed for the anatase–water interface.^78^

The bending vibrations (cf. Figure 5iii) are observed to exist within the frequency range of *grosso modo* 1450 and 1800 cm^–1^ for the bulk water region and lower and upper surfaces. Quite apparently, the bending vibration alternating the valence H–O–H angle of the water molecules is not affected by the applied field, although with the exception of the lower surface, which is observed to be green-shifted toward lower intensity at a peak frequency of ∼1600 cm^–1^ and dark-gold-shifted with the appearance of “wagging modes” to higher intensity at a frequency of 1800 cm^–1^. However, slight suppression of the bond angle bending modes associated with the antiparallel field alignment is attributable to water confinement caused by the relative stability of the hydrogen bonds and respective translational inhibition of the oxygen atoms due to stable electrostatic bonds with the surface iron atoms.^14^

The stretching modes for bulk water and the hematite–water interface are displayed in Figure 5ii.

As evinced, the bulk water region reasonably mimics the experimental O–H stretching band with red shifting of the modes corresponding to zero field to slightly higher frequency from the initial frequency range existing between 2600 and 4000 cm^–1^ and corresponding also to static-field conditions. Inception of the field with varying intensity accentuated the asymmetric stretching band and conversely suppressed the symmetric mode as shown in Figure 5iii. In a similar notion, stretching modes corresponding to the upper surface (parallel alignment) behaved identically to those in the bulk region, which follows accordingly with librational and bending modes reported earlier. Considering the lower surface, distinctive features are seen to emerge with the stretching mode located at a frequency between 2600 and 4000 cm^–1^, similar to upper surface but differing in such a way that an increase in the field intensity of 0.10 V/Å is exhibited by green-elongation and red-broadening of the asymmetry mode with complete suppression of the symmetric mode.

### 3.5 Charge Density

An integral part of this study involved examining notable changes in charge density that serve as characteristic features observed for interfacial systems, particularly in contact with liquid water. The distribution of total charge density and that of the atomic elements in the hematite–water interface are depicted in Figure S10 of the Supporting Information. The work in ref (44) was seminal but omits the charge density of the atomic elements; therefore, in order to obtain the Hirshfeld charge density of the atomic elements in both the bulk and adsorbed water interfaces,^79^ individual atoms were decomposed and Poison’s equation was employed because it provides the relationship between charge density ρ(*z⃗*) in the system and electrostatic potential function φ(*z⃗*). It is instructive to note that due to the scope of this study, the distribution of electrostatic potential was neglected while analyzing the observed changes occurring in close proximity to the surface in relation to the total and electronic (atomic) charge density. More so, the change proceeding along the direction perpendicular to the hematite surface was implemented by the one-dimensional version of Poisson’s equation and defined as
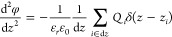
1where the total charge density is represented by the atomic point charges *Q*_*i*_ in order to increase efficiency and reduce the size of the stored data, which led to the charge density ρ(*z⃗*) approximated by a point charge distribution. The permittivity of the environment was expressed as the product of the vacuum permittivity, ε_0_, and relative permittivity of the environment, ε_*r*_. For simplicity, the permittivity in the interfacial region was neglected and the Hirshfeld atomic charges tracked along the NE-AIMD trajectories were used. As clearly depicted from the distribution (cf. Figure S10), one can see that the charge distribution converges to zero in the bulk water region. Obvious variation in the charge density magnitude arises only in close proximity to the surface where adsorbed water is confined and relatively well-organized. For the case of the zero-field condition, atomic field variations corresponding to the surface Fe atom are seen to fluctuate over the distance prior to the boundary of the interfacial region, and at an extended distance, the net charges are seen to balance to zero in the bulk water region, which confirms the polarization of water molecules by the Fe ion, thus making it easier for OH bonds to break. Upon introduction of an electric field, the effect of charge density on the cation is enhanced until a field intensity 0.10 V/Å threshold, also bearing in mind that perhaps a saturation r.m.s intensity has been attained where no further effect will be visible.^35^ The surface oxygen atom is seen to mimic, in the opposite direction, the charge density of the surface Fe atom where increasing the field intensity beyond a certain threshold also resulted in minimal changes in the charge density under the zero-field condition. In spite of the variation in magnitude observed in terms of the atomic charge density in relation to the surface, changes in the atomic charge distribution emphasize *ipso facto* the charges that transpire in the interfacial region, thereby providing a strong indication of the adsorption interaction occurring at the hematite surface, in addition to the typical water layering ascribed to the aqueous/metal-oxide surface. Notwithstanding, total and atomic charge separation persisted for roughly 50% coverage on the hematite surface, which is tantamount to a previous study performed on titania–water interfaces.^66^ However, it should be remarked that despite dogged charge separation, the charge species cannot spontaneously diffuse from the interfacial region as a result of their strong binding to the hematite and electrostatic stabilization that serve to inhibit desorption by a high energy barrier.

**Figure 6:**
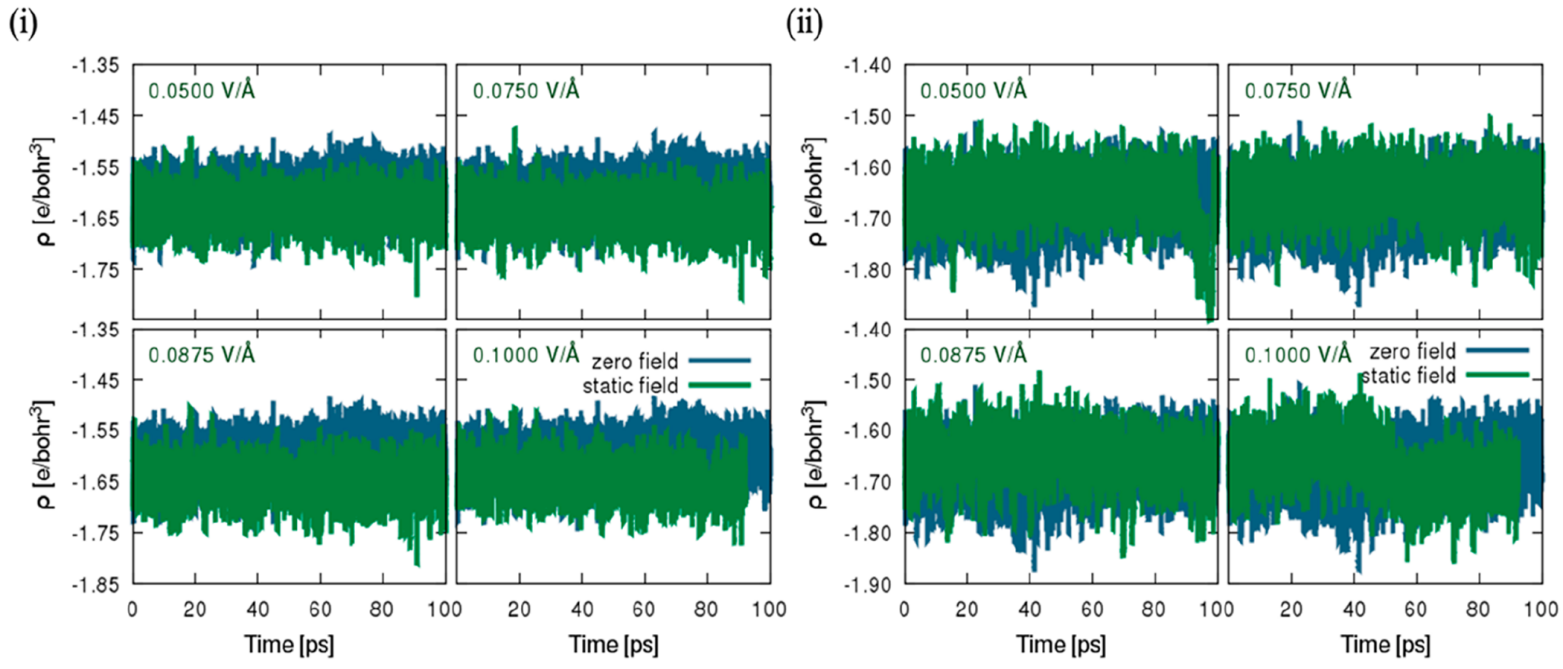
Time distribution of Hirshfeld charge density of the oxygen surface (O_surf_) hematite slab collected during (NE-) AIMD simulation under static field conditions of magnitudes 0.05, 0.075, 0.0875, and 0.10 V/Å in comparison with the zero-field condition of magnitude 0.00 V/Å. The effect of the applied static electric field in either surface direction alignment as depicted in Figure 1 denotes (i) the left-hand side (LHS) as the lower surface wherein the external-field vector is in an antiparallel alignment *vis-à-vis* its local surface normal and (ii) the right-hand side (RHS) represents the upper surface with parallel field-vector alignment.

**Figure 7:**
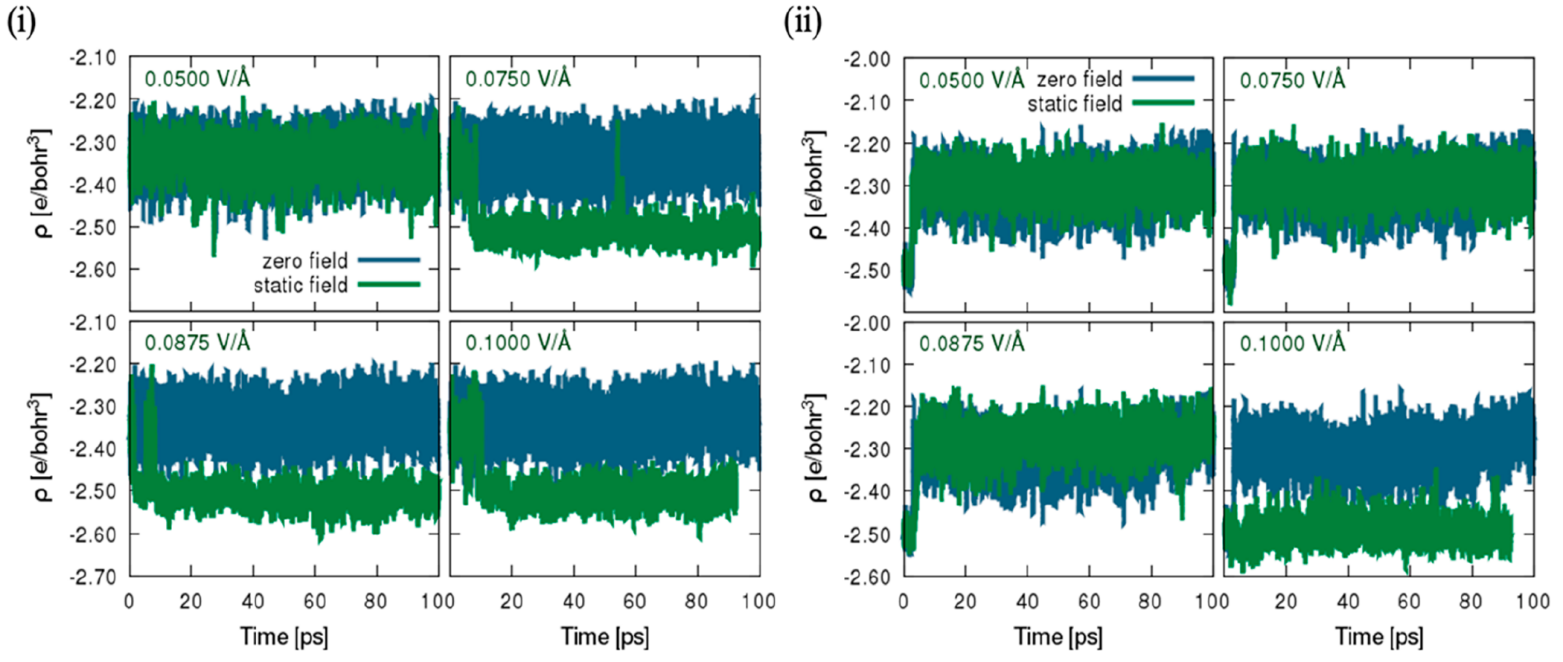
Time distribution of Hirshfeld charge density of the oxygen atoms in the hematite–water interface collected during (NE-) AIMD simulation under static field conditions of magnitudes 0.05, 0.075, 0.0875, and 0.10 V/Å in comparison with the zero-field condition of magnitude 0.00 V/Å. The effect of the applied static electric field in either surface direction alignment as depicted in Figure 1 denotes (i) the left-hand side (LHS) as the lower surface wherein the external-field vector is in an antiparallel alignment *vis-à-vis* its local surface normal and the (ii) right-hand side (RHS) represents the upper surface with parallel field-vector alignment.

Further gauging the plausibility of the occurrence of changes in the Hirshfeld charge density of atomic elements in relation to the measured time in picoseconds located in the bulk water region and aqueous hematite interface collected during (NE-MD) AIMD simulations under both zero field and externally applied field effects with varying magnitude, Figures 6–7 and S11–S13 in the Supporting Information depict in detail the time distribution obtained from the tracked Hirshfeld charges,^79^ which was unexplored in a previous study^44^ and is described in this study, in relation to the deformation density, as the differences between the molecular and unrelaxed atomic charge densities. To avoid any form of ambiguity, in brief, all samples corresponding to the net charges of individual atoms in each snapshot were collected in the trajectory under zero field and an external static field. As shown in Tables 5 and S1–S4 in the Supporting Information, the standard deviation and mean of the Hirshfeld charges relating to individual atom type located in the bulk region and hematite–water interface at the specified field intensity of appropriate time measured in picoseconds after equilibration were calculated. In terms of the hematite slab and a required thorough examination of the total standard deviation and mean (cf. Tables 5 and S1–S4), it is apparent that Hirshfeld charges change minimally with the exception of surface iron atoms at the hematite surface, which are seen to increase with increasing field intensity from zero field to the static field condition, in addition to oxygen atoms that are observed to reduce from a field intensity of 0.05 to 0.10 V/Å. Perhaps it can be presupposed to be arbitrary due to the zero-field value appearing randomly between measured static field values of 0.05 and 0.075 V/Å. Nonetheless, minimal changes are still indicative of charge transfer occurring from water molecules to the surface atom.^80^ Considering the bulk-water phase and antiparallel and parallel surfaces, it is obvious that changes in the Hirshfeld charge values appear to be minimal, which evidently cannot be remarked as corresponding to any form of field increments or reduction hitherto in relation to the standard deviation or mean with varying electric-field intensity. Moreover, it is instructive to note that the analyses were constructed in such a manner that the zero-field conditions in each snapshot of the trajectory were compared with a static field of varying strengths.

**Table 5:** Standard Deviation and Mean of the Hirshfeld Charges of the Individual Atom Types Located in the Hematite/Water Interface at the Specified Static Electric Field Intensity [V/Å] of a Given Time [ps] after Equilibration

				Hirshfeld charges	
field (intensity)	time (ps)	hematite/water interface	atom type	st dev	mean
0.1000	85.9	parallel	Fe-surf	0.04210	2.40630
			O-surf	0.05010	–1.66800
			H_w_	0.01000	0.26370
			O_w_	0.02700	–2.49140
		bulk water	H_w_	0.01120	0.26080
			O_w_	0.02930	–2.50570
		antiparallel	Fe-surf	0.04030	2.35510
			O-surf	0.03570	–1.64490
			H_w_	0.00880	0.25350
			O_w_	0.05150	–2.49480

## Conclusions

In nonequilibrium AIMD, we investigated the presence of water splitting-up on the hematite–water interface under the influence of an externally applied uniform static electric field with increasingly varying strengths. Unarguably, the spontaneous adsorption of water molecules on the hematite surface was observed, which subsequently proceeded with the dissociation of water to hydronium ion, hydroxyl, and proton species that are attached to the surface oxygen and iron, respectively. The application of the field in the direction pointing along the +*z* axis of the supercell served to disrupt the hydrogen-bond network and consequently accelerated the water splitting reaction, particularly when the external applied static field (of varying intensity) was efficiently applied in the direction of the oxide along the +*z* axis of the vector in the lower surface (i.e., antiparallel alignment) of the hematite slab. In as much as the Grotthuss hopping mechanism effect was expatiated with increasing strength beyond a field intensity of 0.05 V/Å, under the zero-field condition, the water molecules were seen to be chemisorbed on the hematite surface and thus resulted in the rapid dissociation of water molecules to H^+^ and OH^–^ species.

Furthermore, we demonstrated a clear indication of increased perturbation in the intramolecular geometry corresponding to the hematite–water interface in comparison to those in the water–water surfaces interaction and bulk water region. In general, we also reported H-bond lengths shifting slightly toward larger values in the upper surface in comparison to those in the lower surface while the reverse effect was observed for the H-bond angles. Clear correlation existing among the number of hydrogen bonds, H-bond angles, and H-bond lengths was established, with the average number of hydrogen bonds reducing from the upper to lower surface in the case of the water–water interaction while beyond a certain reduced field strength (e.g., 0.05 V/Å) threshold, the average number of hydrogen bonds decreased in the hematite–water interface. In addition, we also confirmed a notable relationship existing between the hydrogen-bond strength and hydrogen-bond lifetime where stronger (shorter) hydrogen bonds were reported to dissociate slowly due to an extended persistence time required for breakage and reformation events as a result of increased bond dissociation energy. Moreover, interesting yet intriguing evidence of the characteristic features associated with three band modes was showcased by Fourier transforming the velocity autocorrelation function of the hydrogen atoms. Similar trends were observed for bulk water and the upper surface in terms of the librational and bending vibrations. The librational modes were both unaffected by the applied field, whereas the lower surface promoted the evidence of the Grotthuss mechanism, particularly at higher field intensity with less scope for rotation and oscillating motion due to confinement and suppression effects. The presence of wagging modes was observed to be pronounced in the lower surface with the occurrence of the H–O–H bending vibration. The accentuation of asymmetric stretching modes in the presence of a field with varying strengths was observed with subsequent suppression of the symmetric modes exhibited by the O–H stretching band of the bulk water region and hematite–water interface. Moreover, variation in the total and atomic charge density magnitudes obtained by Poisson’s equation occurred in close proximity to the surface where adsorbed water is confined and relatively well-organized. Meanwhile, the Hirshfeld charge density^79^ of atomic elements in relation to the measured time in picoseconds opens new vistas in assessing the plausibility of the occurrence of charge transfer from water molecules to the surface atoms.

**Figure 8:**
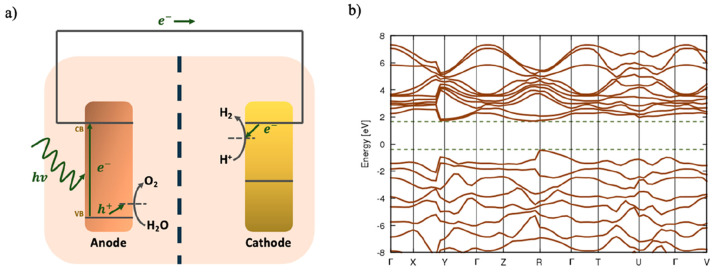
(a) Diagrammatic illustration of the PEC cell for the water-splitting process. As depicted, conduction and valence band edges of the semiconducting anode and cathode are shown by the upper and lower horizontal solid lines, while the horizontal dashed lines represent energy levels of the H_2_O/O_2_ and H_2_/H^+^ redox couples. (b) Band structure of pristine hematite plotted along the path in the Brillouin zone as suggested by the SeeK-path program.^83^ The Fermi level is set to energy zero.

At this stage, it is clear that NE-AIMD results have informed the general mechanics of water photodissociation at the hematite surface, and as such, it is fitting to remark succinctly that while the photoexcited electrons and holes are produced from the absorption of light in the hematite, an oxidation–reduction reaction couple transpires at the electrode surface (cf. Figure 8), with the holes reacting with water molecules in the oxygen-evolution reaction at the anode (i.e., hematite–water interface)^81,82^ as
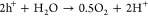
2
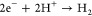
3

In this broader PEC and electrochemistry context, the results of this study inform us of the first oxidation process with an electrochemical potential of 1.23 V taking place at the hematite–water interface. Figure 8b shows that the computed indirect band gap of the pristine hematite is 2.02 eV, where the conduction band minimum (CBM) lies at the Y point and the valence band maximum (VBM) lies between R and the gamma point. The value slightly underestimates the experimental value ranging between 2.1 and 2.3 eV^3,39^ but is in close agreement with the theoretical value of 2.05 eV reported in ref (84). In terms of Fe–O interactions, both water (oxygen atom) and hydroxyl ions were attracted (i.e., electrostatic force of attraction or repulsion between the charged particles) to Fe ions in order to form the required physical absorption that could effectively facilitate reaction with the photoexcited holes that had diffused through the hematite surface. Once the external field applied was strong enough to induce further dissociation, it was observed and theorized that the splitting process proceeded with a series of proton jumps from the newly formed hydronium to the next H-bonded neutral molecules along the field direction, thus enabling the formation of another hydronium from which a different proton hops and jumps, and the process continues in such manner (cf. Figure 8a). Given that ionic diffusion and consequently conduction are activated by the dissociation process, the occurrence of fields evidently breaks the symmetry of physical space, thus providing the spatial means required for the proton-hopping manifestation. A series of these equilibrium reactions are described as follows
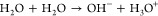
4in which the right-directed field alignment (cf. Figure 1) induces a proton jump from the left to right water molecules and conversely after the dissociation process, expected recombination reactions transpire as required
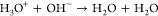
5where the extra proton originates from a hydronium in the opposite direction of the hydroxide with respect to the field.

Ostensibly, the results presented in the current study have not only described the field-enhanced dissociation of hematite-bound water molecules *per se* but also further account for the diffusion and rearrangement of hydroxyl ions species, VDOS, intramolecular geometry, intermolecular structures, and charge density.

## Supporting Information Available

Snapshot of the horizontal model with indicated interface and bulk-water regions (Figure S1); zero-field RDF and coordination number (Figure S2) and static-field RDF and coordination number (Figures S3–S5); hydrogen-bond lengths, angles, average number of hydrogen bonds, and lifetime (Figures S6–S9); atomic charge density (Figure S10); time distribution of the Hirshfeld charge density in the hematite slab and the bulk water region and hematite–water interface (Figures S12 and S13) (PDF)
