# Supplementary material for: Nonequilibrium Ab Initio Molecular Dynamics Simulation of Water Splitting at Fe2O3–Hematite/Water Interfaces in an External Electric Field
Source: J Phys Chem C Nanomater Interfaces. 2023 Dec 11;127(50):24088–105. doi: 10.1021/acs.jpcc.3c05119 (PMC10749450; doi:10.1021/acs.jpcc.3c05119)
Supplement: Supplementary file 1 — jp3c05119_si_001.pdf [file jp3c05119_si_001.pdf]

Supplementary Information
For
Non-Equilibrium *Ab-Initio* Molecular Dynamics Simulation Of Water Splitting At Fe_2O_3-Hematite/Water Interfaces In External Electric Field

Mary T. Ajide and Niall J. English*
School of Chemical & Bioprocess Engineering, University College Dublin, Belfield, Dublin 4, Ireland
* Corr. author – niall.english@ucd.ie

The structural and hydrogen-bond analysis implemented for the bulk and adsorbed water layer were analysed by calculating the H_w−O_w−H_w valence angles, water O_w−H_w bond lengths, and individual-water dipoles as mean value integration of $\langle x \rangle = \int xP(x)dx$ of the corresponding normalised histograms $P(x)$ obtained from the simulation. Moreover, the respective histograms depicting hydrogen bond analysis; hydrogen-bond angles, lengths, hydrogen bonds number are presented therein. The hydrogen bond were selected on the geometry criteria for arrangement of donor (D), acceptor (A), and the hydrogen (H). Classifying the hydrogen bond arrangement was predicated on maximum A−D distance set at 3.5 Å and while A−D−H angle was required not to exceed 35°. Furthermore, dynamics of the hydrogen bonds was described using Luzar-Chandler model[1] for predicting average hydrogen-bond lifetime.

The vibrational density of state (VDOS) for zero-field and external electric-fields implemented was obtained by Fourier-transforming the velocity autocorrelation function of hydrogen atoms.

$$I(\omega) \propto \int \langle \vec{v}_H(t_0 + t) \rangle e^{-i\omega t} dt \qquad (1)$$

The relaxation time of hydrogen bonds was characterised using an autocorrelation function $c(t)$[1-2]

$$c(t) = \frac{\langle h(0)h(t) \rangle}{\langle h(0)h(0) \rangle} \qquad (2)$$

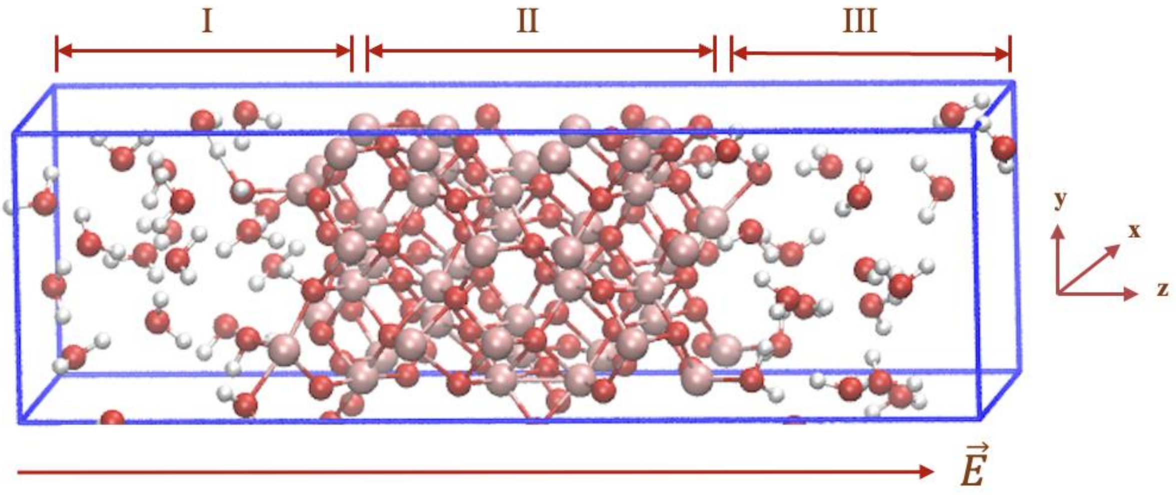

Figure S1. Representative model of the aqueous hematite interface implemented for the calculation. The snapshot of the supercell was performed under periodic boundary condition (PBC) after zero-field conditions equilibration. As clearly shown, the direction of the applied static electric field E is indicated by the darkish orange arrows, pointing along the + z-axis of both supercell. I) The left-hand side (LHS) denotes the lower surface wherein the external-field vector is in antiparallel alignment *vis-à-vis* its local surface normal. II) The hematite slab located at the central part of the supercell where iron (Fe) atoms are shown in pink colour, and oxygen are shown in red. III) The right-hand side (RHS) denotes the upper surface with parallel field-vector alignment. The water molecules where oxygen are depicted in red and hydrogen as white, fills the supercell on both sides, under which the period boundary conditions are imposed.

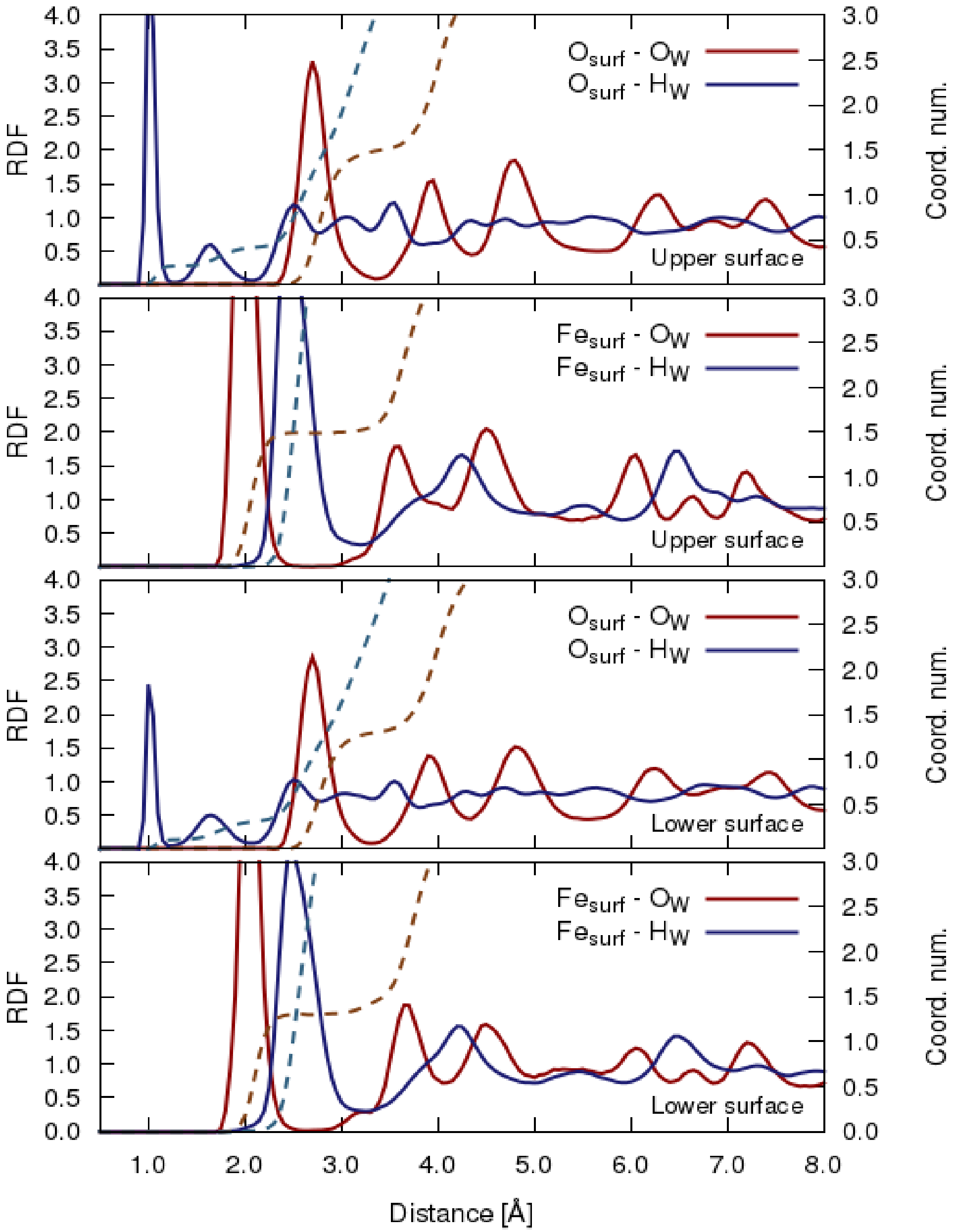

Figure S2. Radial distribution functions (RDF) detecting interaction of hematite surface iron (Fe_{surf}) and oxygen (O_{surf}) atoms with hydrogen (H_W) and water oxygen (O_W) under zero-field condition for both surface direction alignment as depicted in Figure 1 denoting the left-hand side (LHS) as the lower surface wherein the external-field vector is in antiparallel alignment *vis-à-vis* its local surface normal and the right-hand side (RHS) denoting the upper surface with parallel field-vector alignment.

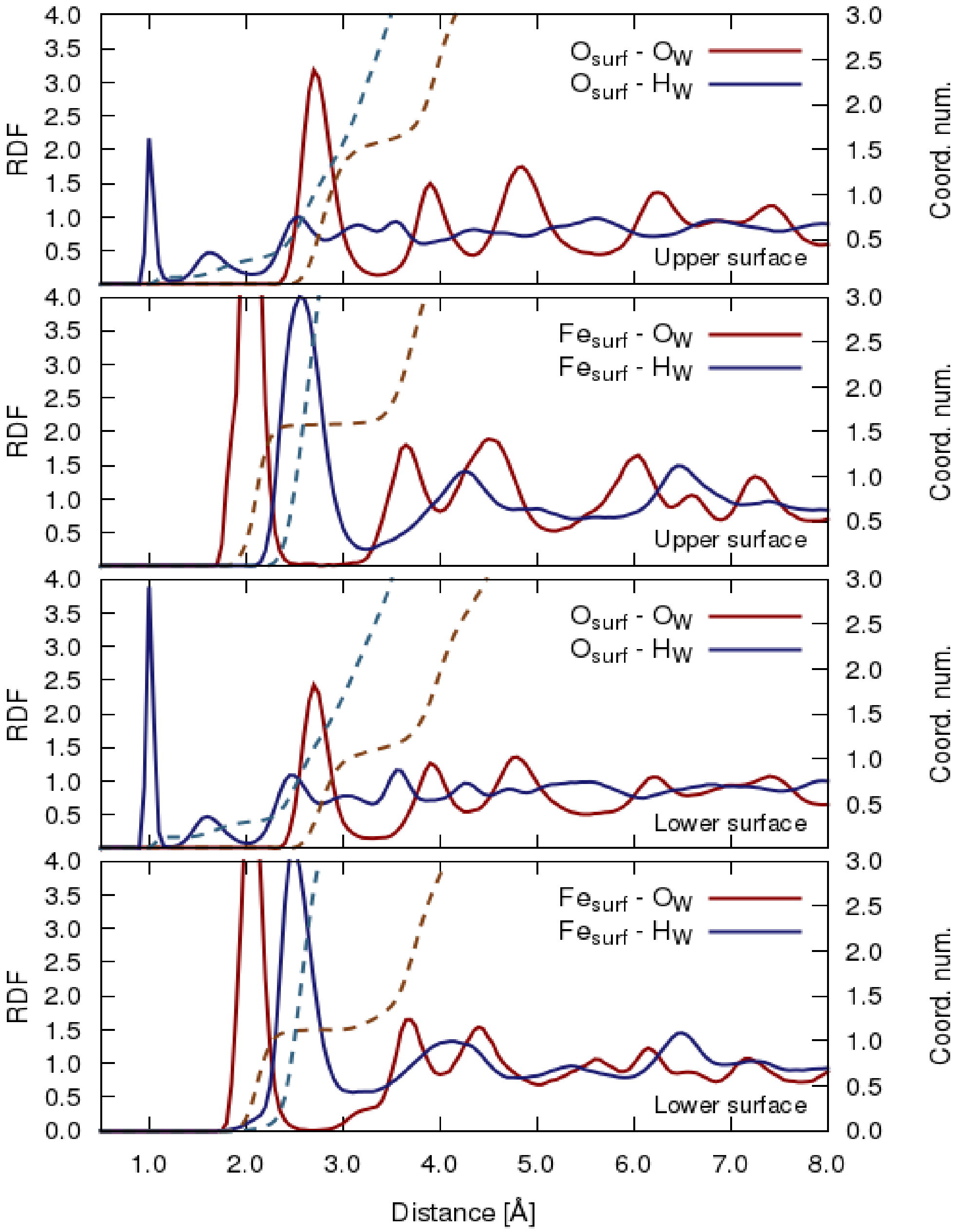

Figure S3. Radial distribution functions (RDF) detecting interaction of hematite surface iron (Fe_{surf}) and oxygen (O_{surf}) atoms with hydrogen (H_W) and water oxygen (O_W) under external static electric field of 0.05 $V/Å$ for both surface direction alignment as depicted in Figure 1 denoting the left-hand side (LHS) as the lower surface wherein the external-field vector is in antiparallel alignment *vis-à-vis* its local surface normal and the right-hand side (RHS) denoting the upper surface with parallel field-vector alignment.

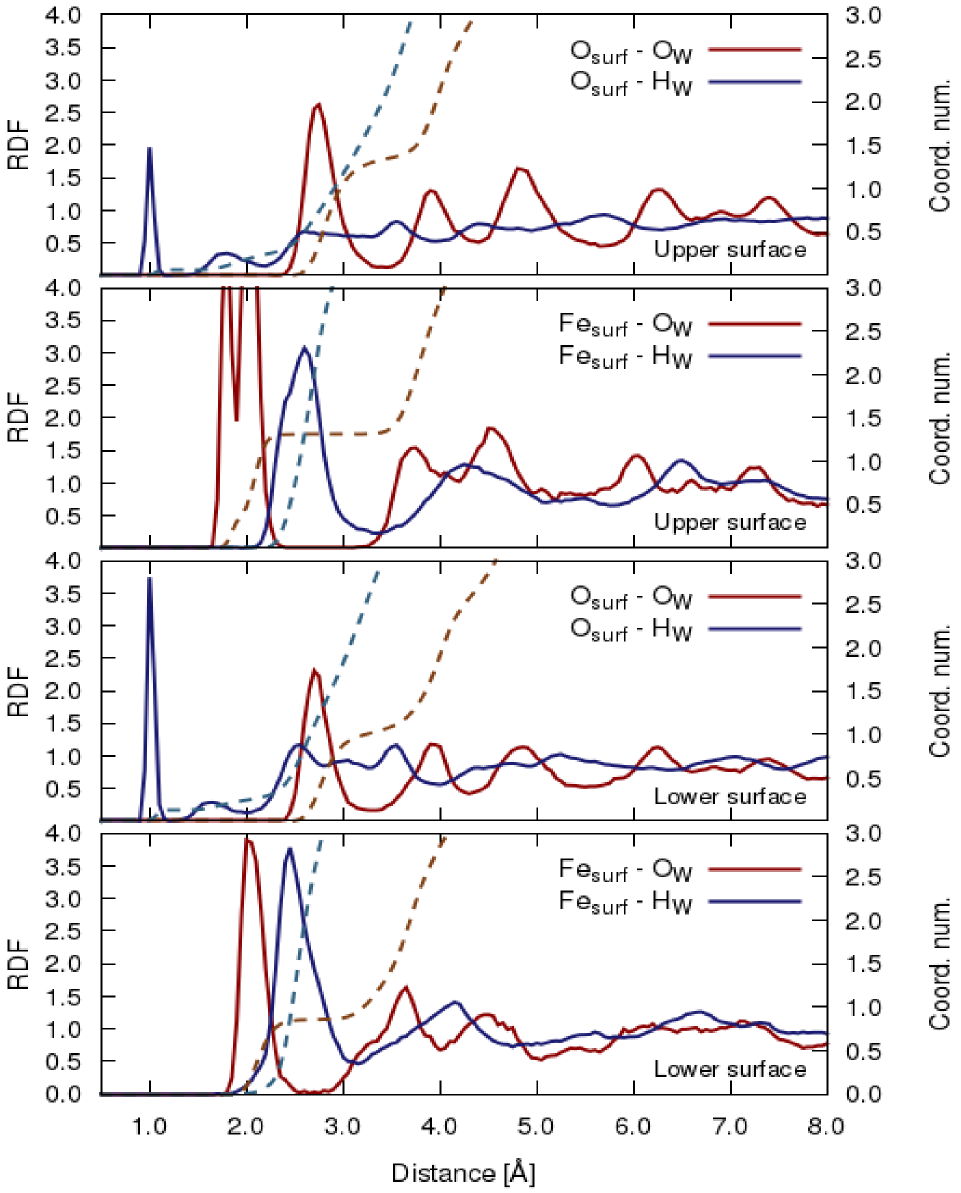

Figure S4. Radial distribution functions (RDF) detecting interaction of hematite surface iron (Fe_{surf}) and oxygen (O_{surf}) atoms with hydrogen (H_W) and water oxygen (O_W) under external static electric field of 0.075 $V/Å$ for both surface direction alignment as depicted in Figure 1 denoting the left-hand side (LHS) as the lower surface wherein the external-field vector is in antiparallel alignment *vis-à-vis* its local surface normal and the right-hand side (RHS) denoting the upper surface with parallel field-vector alignment.

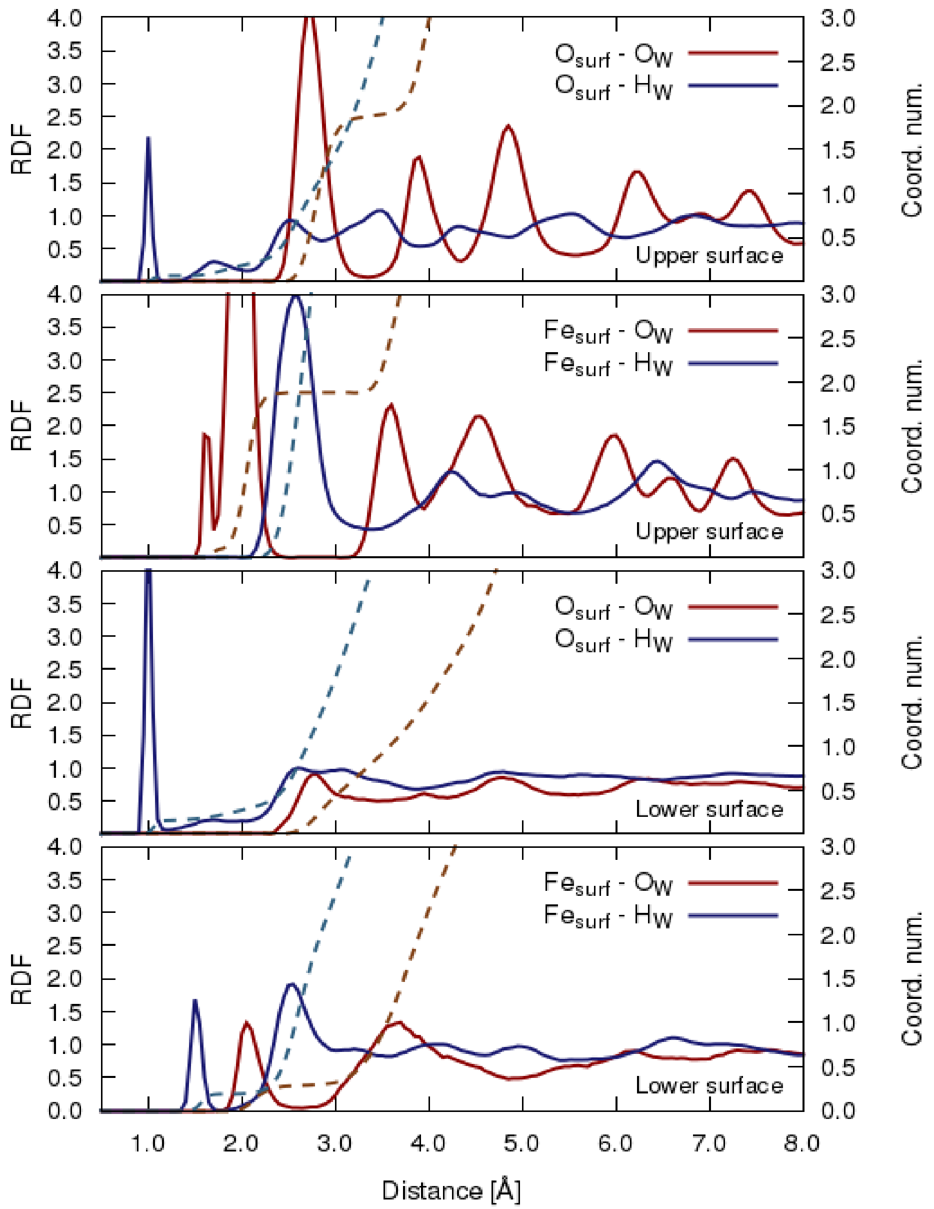

Figure S5. Radial distribution functions (RDF) detecting interaction of hematite surface iron (Fe_{surf}) and oxygen (O_{surf}) atoms with hydrogen (H_W) and water oxygen (O_W) under external static electric field of 0.0875 $V/$Å for both surface direction alignment as depicted in Figure 1 denoting the left-hand side (LHS) as the lower surface wherein the external-field vector is in antiparallel alignment *vis-à-vis* its local surface normal and the right-hand side (RHS) denoting the upper surface with parallel field-vector alignment.

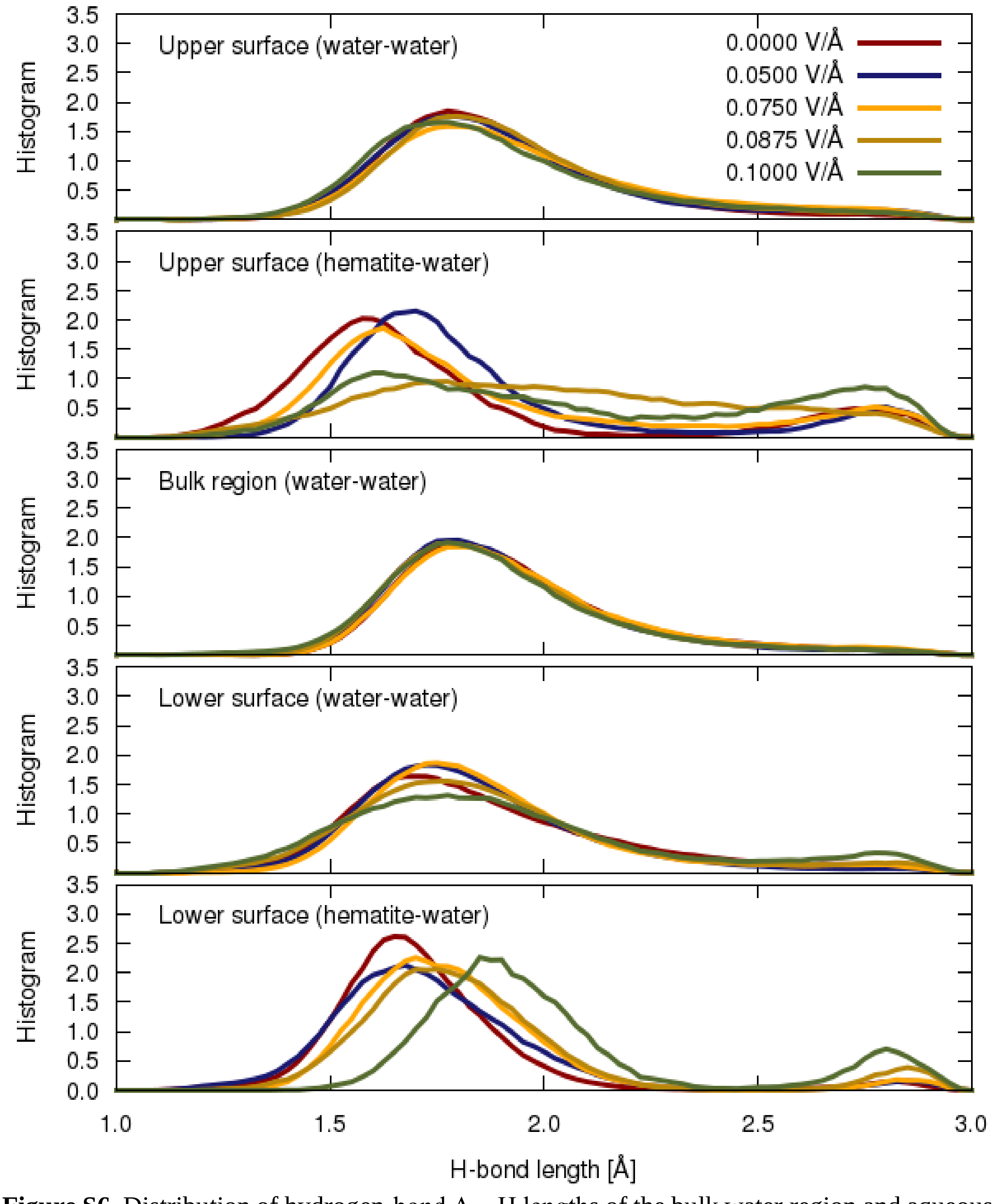

Figure S6. Distribution of hydrogen-bond A—H lengths of the bulk water region and aqueous hematite interfaces under the effect of applied static electric field in either surface direction alignment as depicted in Figure 1 denoting the left-hand side (LHS) as the lower surface wherein the external-field vector is in antiparallel alignment *vis-à-vis* its local surface normal and the right-hand side (RHS) denoting the upper surface with parallel field-vector alignment

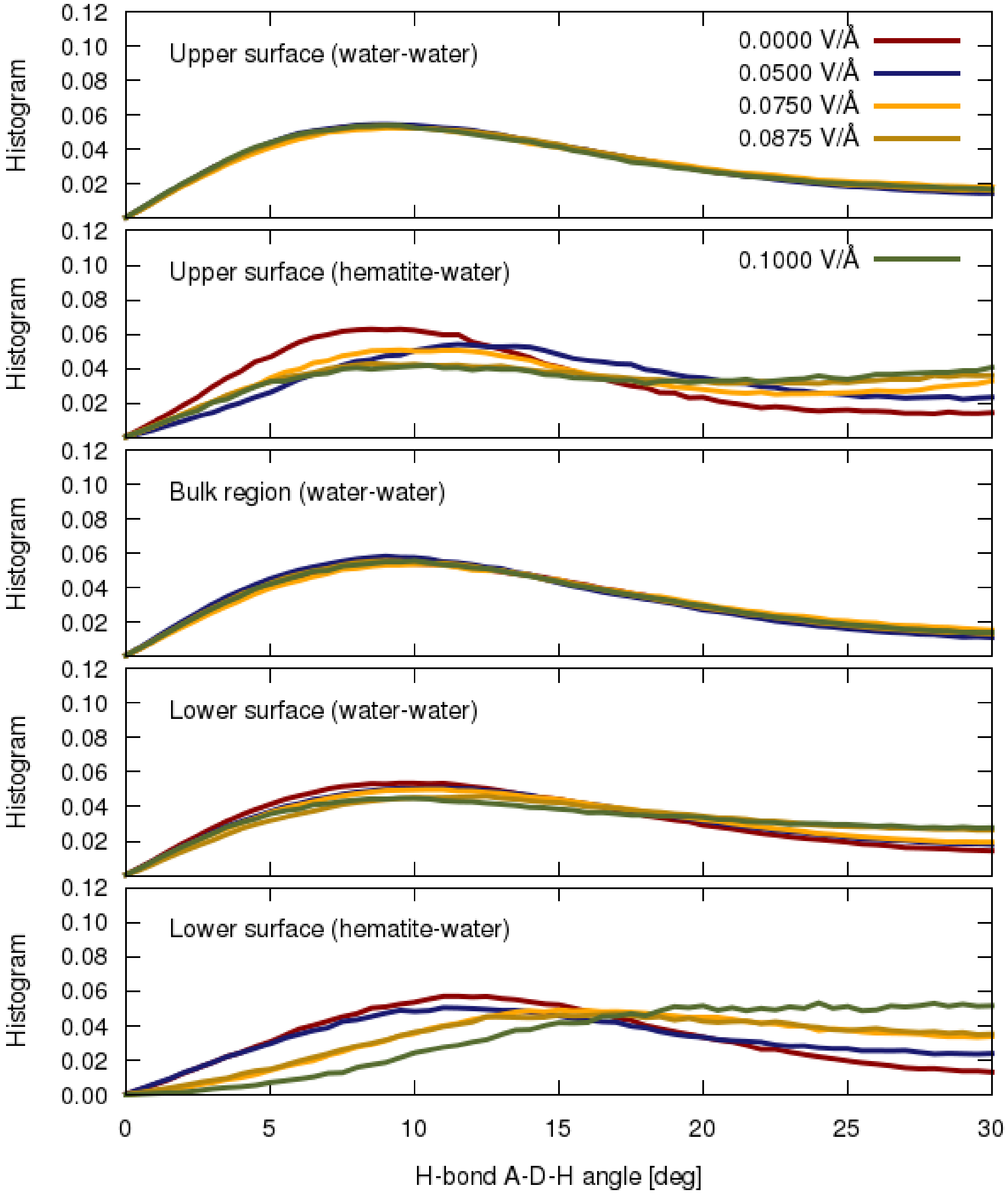

Figure S7. Distribution of hydrogen bond angles of the bulk water region and aqueous hematite interfaces under the effect of applied static electric field in either surface direction alignment as depicted in Figure 1 denoting the left-hand side (LHS) as the lower surface wherein the external-field vector is in antiparallel alignment *vis-à-vis* its local surface normal and the right-hand side (RHS) denoting the upper surface with parallel field-vector alignment.

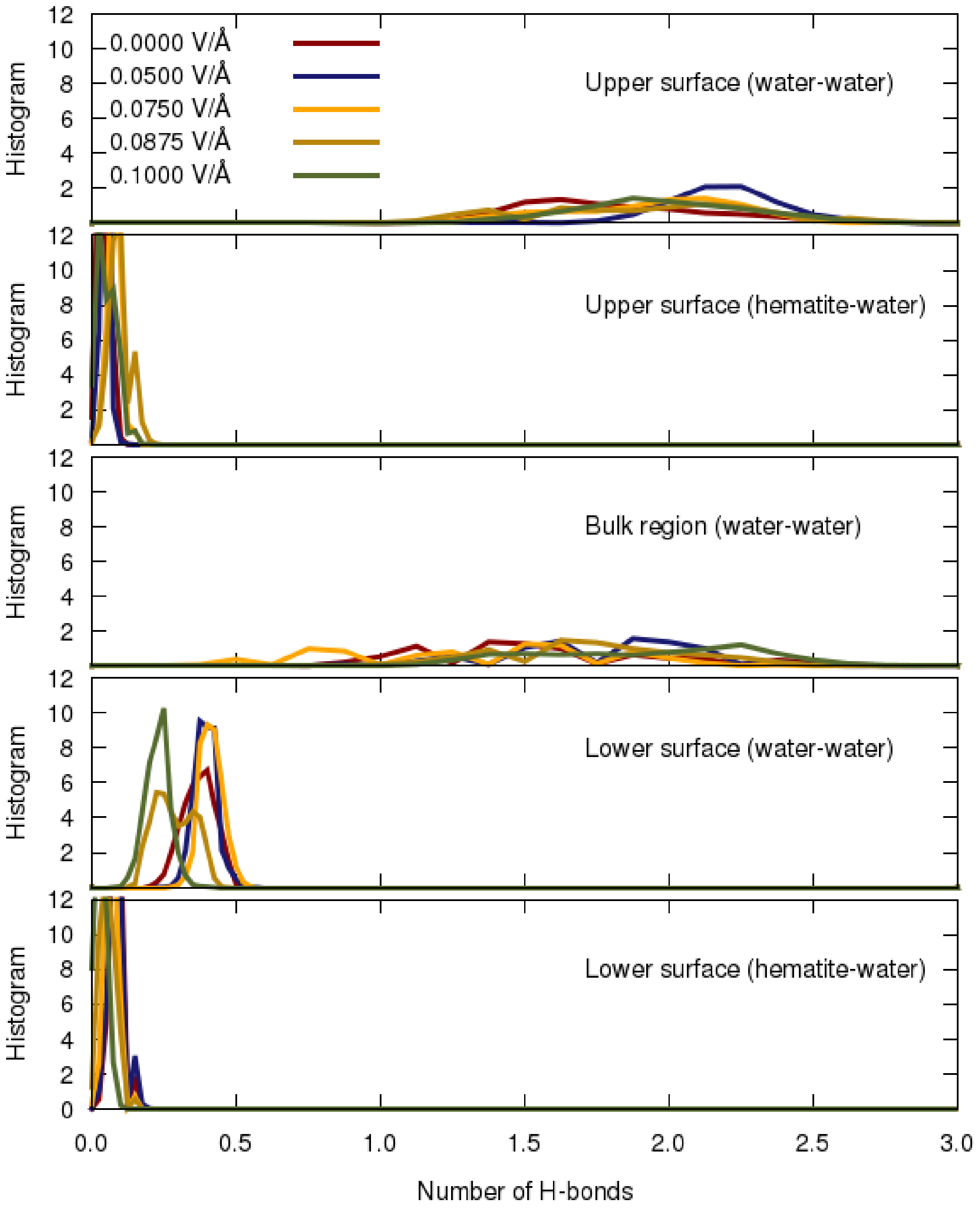

Figure S8. Distribution of average number of hydrogen bonds per water molecule in the bulk water region and aqueous hematite interfaces under the effect of applied static electric field in either surface direction alignment as depicted in Figure 1 denoting the left-hand side (LHS) as the lower surface wherein the external-field vector is in antiparallel alignment *vis-à-vis* its local surface normal and the right-hand side (RHS) denoting the upper surface with parallel field-vector alignment.

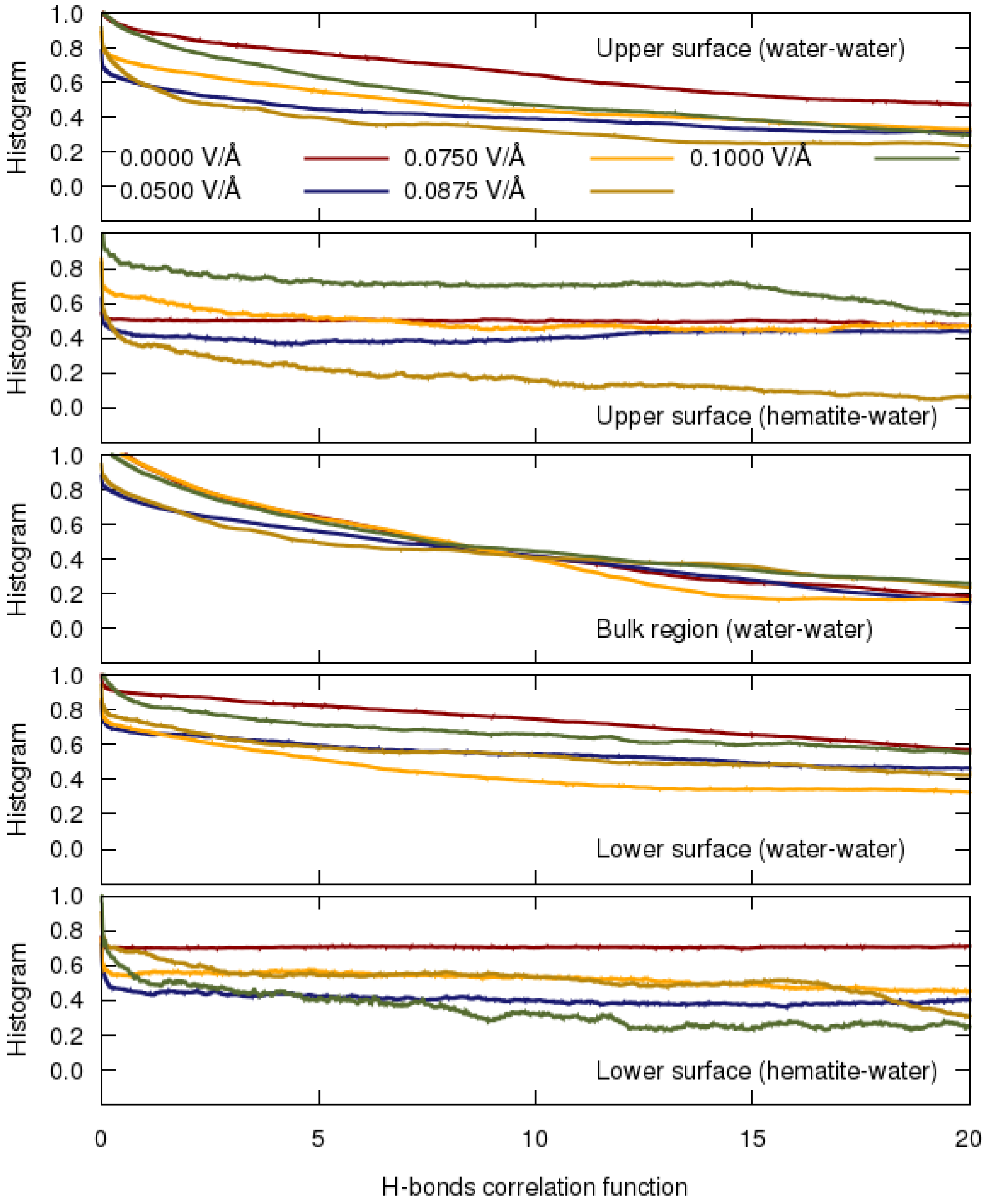

Figure S9. Hydrogen-bond lifetime correlation function of the bulk water region and aqueous hematite interfaces under the effect of applied static electric field in either surface direction alignment as depicted in Figure 1 denoting the left-hand side (LHS) as the lower surface wherein the external-field vector is in antiparallel alignment *vis-à-vis* its local surface normal and the right-hand side (RHS) denoting the upper surface with parallel field-vector alignment.

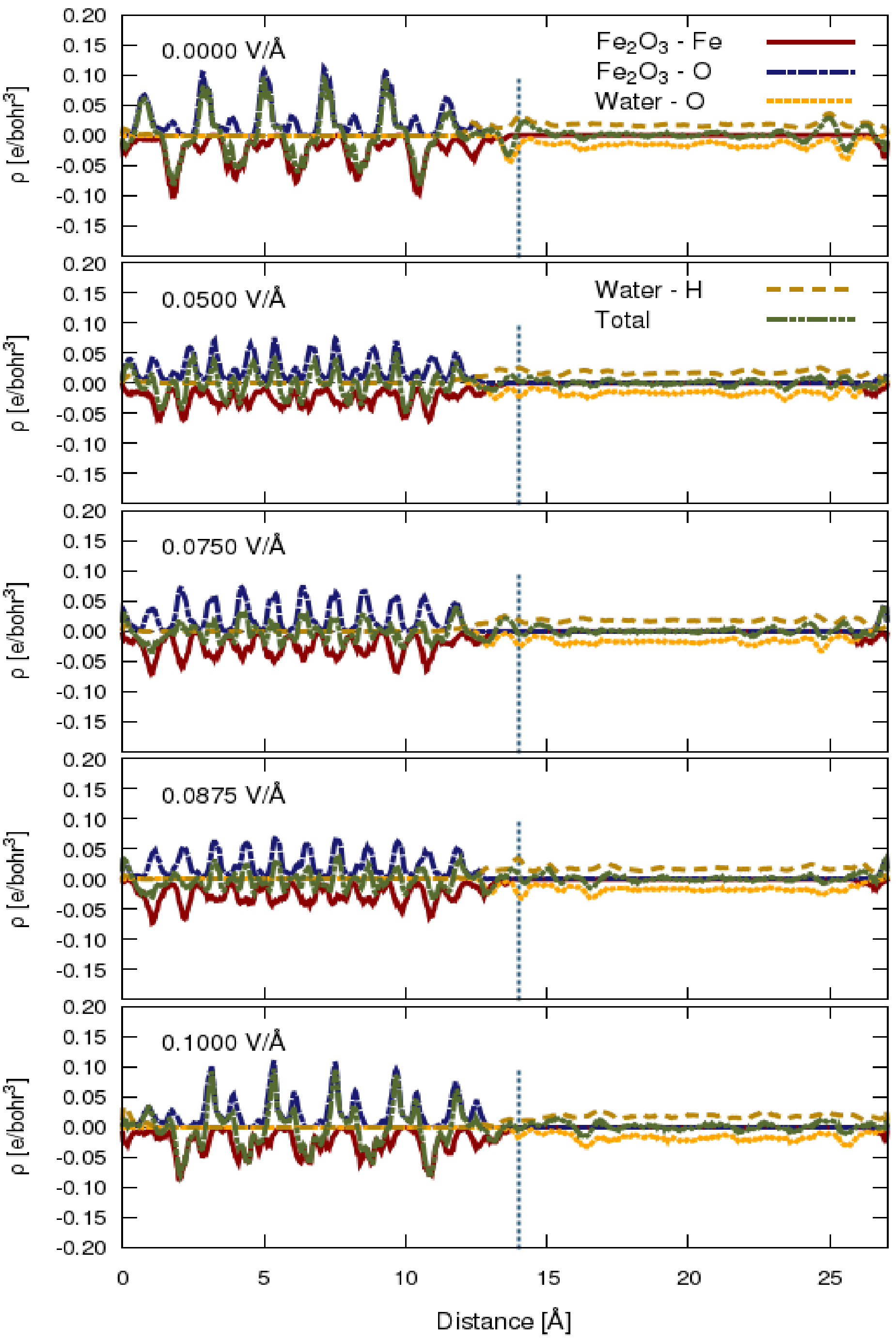

Figure S10. Atomic charge density $\rho(z)$ distribution in the hematite surface iron (Fe_{surf}), oxygen (O_{surf}) atoms, hydrogen (H_W) and water oxygen (O_W) of the supercell collected during (NE-)AIMD under externally applied static field. The measured distances was calculated from the average position of the outermost Fe-atom layer on the corresponding hematite surface. As depicted, the dotted blue lines indicates the *de facto* boundary of the interfacial region and the direction of applied electric field follows accordingly as described in Figure 1.

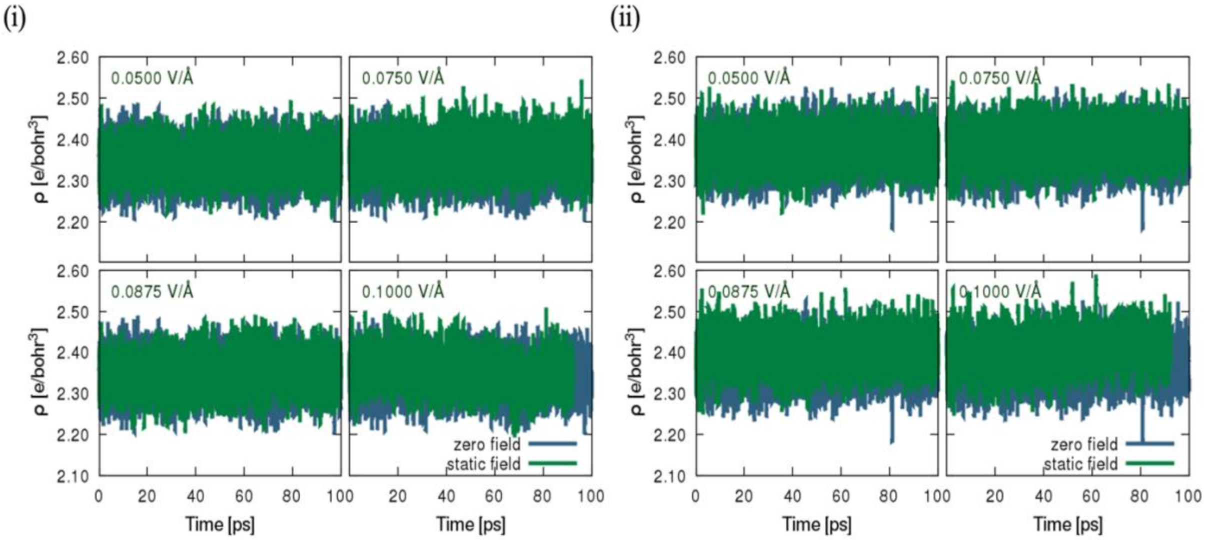

Figure S11. Time distribution of Hirshfeld charge density of the surface iron (Fe_{surf}) hematite slab collected during (NE-) AIMD simulation under static field conditions of magnitudes 0.05 $V/\text{Å}$, 0.075 $V/\text{Å}$, 0.0875 $V/\text{Å}$ and 0.10 $V/\text{Å}$ in comparison with zero-field condition of magnitude 0.00 $V/\text{Å}$. The effect of applied static electric field in either surface direction alignment as depicted in Figure 1 denotes (i) the left-hand side (LHS) as the lower surface wherein the external-field vector is in antiparallel alignment *vis-à-vis* its local surface normal and the (ii) right-hand side (RHS) represents the upper surface with parallel field-vector alignment.

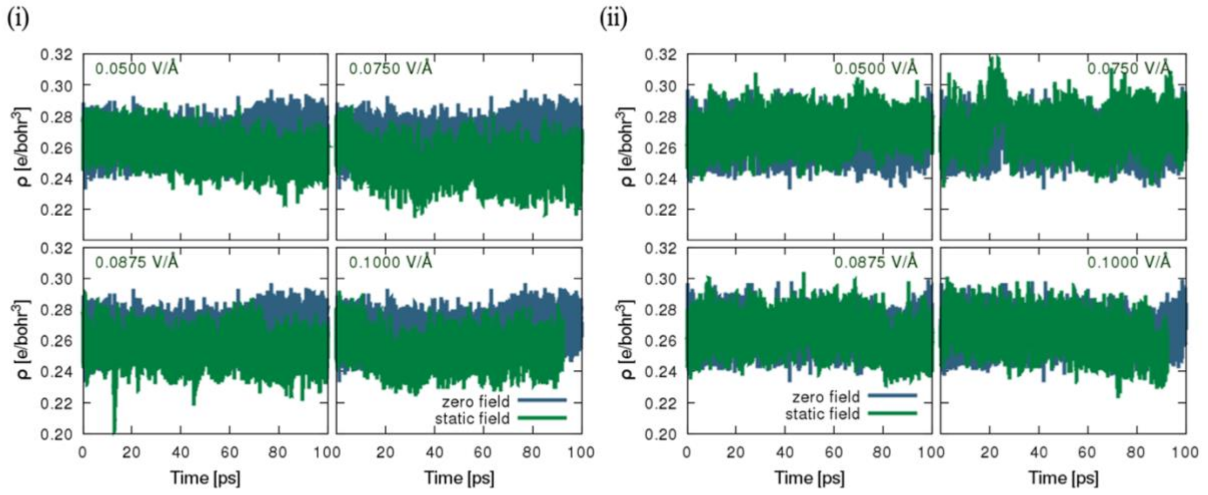

Figure S12. Time distribution of Hirshfeld charge density of the hydrogen water (H_w) in the hematite-water interface collected during (NE-) AIMD simulation under static field conditions of magnitudes 0.05 $V/\text{Å}$, 0.075 $V/\text{Å}$, 0.0875 $V/\text{Å}$ and 0.10 $V/\text{Å}$ in comparison with zero-field condition of magnitude 0.00 $V/\text{Å}$. The effect of applied static electric field in either surface direction alignment as depicted in Figure 1 denotes (i) the left-hand side (LHS) as the lower surface wherein the external-field vector is in antiparallel alignment *vis-à-vis* its local surface normal and the (ii) right-hand side (RHS) represents the upper surface with parallel field-vector alignment.

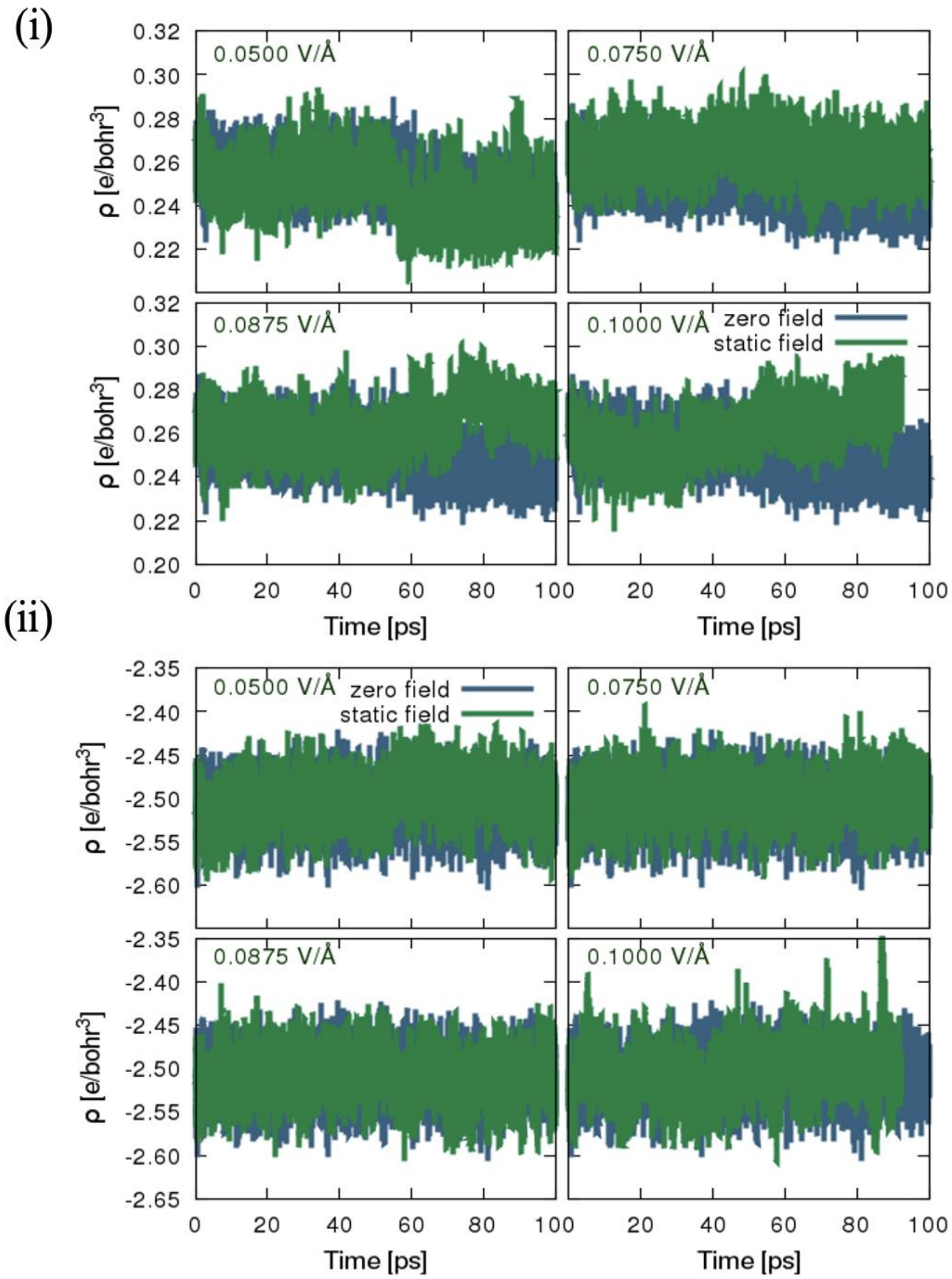

Figure S13. Time distribution of Hirshfeld charge density of the (i) hydrogen water (H_w) and (ii) oxygen atoms in the bulk water region collected during (NE-) AIMD simulation under static field conditions of magnitudes 0.05 $V/Å$, 0.075 $V/Å$, 0.0875 $V/Å$ and 0.10 $V/Å$ in comparison with zero-field spectrum of magnitude 0.00 $V/Å$.

Table S1: Standard Deviation and Mean of the Hirshfeld charges of the individual atom type located in the Hematite/Water Interface at Zero Electric Field Intensity $V/Å$ of Given Time [ps] after equilibration

**Fields (Intensity)**	**Time (ps)**	**Hematite/Water Interface**	**Atom type**	**Hirshfeld charges**	
				**stdev**	**mean**
0.0000	251.8	Parallel	Fe-surf	0.04100	2.37420
			O-surf	0.04400	-1.67220
			Hw	0.00800	0.26560
			Ow	0.05370	-2.31250
		Bulk water	Hw	0.00890	0.25140
			Ow	0.02350	-2.51100
		Antiparallel	Fe-surf	0.03930	2.34360
			O-surf	0.03520	-1.60350
			Hw	0.00760	0.26520
			Ow	0.04420	-2.33910

Table S2: Standard Deviation and Mean of the Hirshfeld charges of the individual atom type located in the Hematite/Water Interface at the specified Static Electric Field Intensity [$V/Å$] of Given Time [ps] after equilibration

**Fields (Intensity)**	**Time (ps)**	**Hematite/Water Interface**	**Atom type**	**Hirshfeld charges**	
				**stdev**	**mean**
0.0500	171.1	Parallel	Fe-surf	0.04080	2.37860
			O-surf	0.08460	-1.69680
			Hw	0.00790	0.27168
			Ow	0.04450	-2.30730
		Bulk water	Hw	0.01250	0.24380
			Ow	0.02520	-2.49930
		Antiparallel	Fe-surf	0.03890	2.35110
			O-surf	0.03600	-1.63360

			Hw	0.00800	0.25560
			Ow	0.04540	-2.34600

Table S3: Standard Deviation and Mean of the Hirshfeld charges of the individual atom type located in the Hematite/Water Interface at the specified Static Electric Field Intensity $[V/Å]$ of Given Time [ps] after equilibration

**Fields (Intensity)**	**Time (ps)**	**Hematite/Water Interface**	**Atom type**	**Hirshfeld charges**	
				**stdev**	**mean**
0.0750	172.7	Parallel	Fe-surf	0.04290	2.39470
			O-surf	0.04950	-1.65470
			Hw	0.01010	0.27150
			Ow	0.04690	-2.29860
		Bulk water	Hw	0.01040	0.26070
			Ow	0.02510	-2.49900
		Antiparallel	Fe-surf	0.04220	2.35910
			O-surf	0.03700	-1.64110
			Hw	0.01320	0.25650
			Ow	0.08750	-2.43750

Table S4: Standard Deviation and Mean of the Hirshfeld charges of the individual atom type located in the Hematite/Water Interface at the specified Static Electric Field Intensity $[V/Å]$ of Given Time [ps] after equilibration

**Fields (Intensity)**	**Time (ps)**	**Hematite/Water Interface**	**Atom type**	**Hirshfeld charges**	
				**stdev**	**mean**
0.0875	203.1	Parallel	Fe-surf	0.04170	2.40140
			O-surf	0.04620	-1.65840
			Hw	0.01010	0.26160
			Ow	0.05080	-2.28050
		Bulk water	Hw	0.00910	0.26330

			Ow	0.02460	-2.50980
		Antiparallel	Fe-surf	0.04190	2.35050
			O-surf	0.03690	-1.65090
			Hw	0.00970	0.25660
			Ow	0.03480	-2.51440

References

(1) Luzar, A.; Chandler, D. Hydrogen-Bond Kinetics in Liquid Water. *Nature* **1996**, 379 (6560), 55–57.

(2) Stillinger, F. H. Theory and Molecular Models for Water. *Adv. Chem. Phys*. **1975**, 1–101.
